# Restoring the FOXO1 geroprotective pathway via seno-resistant mesenchymal progenitor cells alleviates primate epididymal aging

**DOI:** 10.1093/procel/pwag020

**Published:** 2026-03-24

**Authors:** Huifen Lu, Linguo Cai, DongLiang Lv, Guoqiang Sun, Jinghui Lei, Taixin Ning, Zijuan Xin, Haoyan Huang, Ying Jing, Daoyuan Huang, Shuhui Sun, Shuai Ma, Weiqi Zhang, Fei Gao, Rui Chen, Yingying Qin, Weihong Song, Andy Peng Xiang, Juan Carlos Izpisua Belmonte, Guang-Hui Liu, Jing Qu, Si Wang

**Affiliations:** Advanced Innovation Center for Human Brain Protection, National Clinical Research Center for Geriatric Disorders, Aging Translational Medicine Center, Beijing Municipal Geriatric Medical Research Center, Beijing Key Laboratory of Environment and Aging, Xuanwu Hospital Capital Medical University, Beijing 100053, China; Advanced Innovation Center for Human Brain Protection, National Clinical Research Center for Geriatric Disorders, Aging Translational Medicine Center, Beijing Municipal Geriatric Medical Research Center, Beijing Key Laboratory of Environment and Aging, Xuanwu Hospital Capital Medical University, Beijing 100053, China; State Key Laboratory of Organ Regeneration and Reconstruction, Institute of Zoology, Chinese Academy of Sciences, Beijing 100101, China; University of Chinese Academy of Sciences, Beijing 100049, China; State Key Laboratory of Organ Regeneration and Reconstruction, Institute of Zoology, Chinese Academy of Sciences, Beijing 100101, China; Advanced Innovation Center for Human Brain Protection, National Clinical Research Center for Geriatric Disorders, Aging Translational Medicine Center, Beijing Municipal Geriatric Medical Research Center, Beijing Key Laboratory of Environment and Aging, Xuanwu Hospital Capital Medical University, Beijing 100053, China; Advanced Innovation Center for Human Brain Protection, National Clinical Research Center for Geriatric Disorders, Aging Translational Medicine Center, Beijing Municipal Geriatric Medical Research Center, Beijing Key Laboratory of Environment and Aging, Xuanwu Hospital Capital Medical University, Beijing 100053, China; Advanced Innovation Center for Human Brain Protection, National Clinical Research Center for Geriatric Disorders, Aging Translational Medicine Center, Beijing Municipal Geriatric Medical Research Center, Beijing Key Laboratory of Environment and Aging, Xuanwu Hospital Capital Medical University, Beijing 100053, China; Advanced Innovation Center for Human Brain Protection, National Clinical Research Center for Geriatric Disorders, Aging Translational Medicine Center, Beijing Municipal Geriatric Medical Research Center, Beijing Key Laboratory of Environment and Aging, Xuanwu Hospital Capital Medical University, Beijing 100053, China; Advanced Innovation Center for Human Brain Protection, National Clinical Research Center for Geriatric Disorders, Aging Translational Medicine Center, Beijing Municipal Geriatric Medical Research Center, Beijing Key Laboratory of Environment and Aging, Xuanwu Hospital Capital Medical University, Beijing 100053, China; Advanced Innovation Center for Human Brain Protection, National Clinical Research Center for Geriatric Disorders, Aging Translational Medicine Center, Beijing Municipal Geriatric Medical Research Center, Beijing Key Laboratory of Environment and Aging, Xuanwu Hospital Capital Medical University, Beijing 100053, China; Beijing Institute of Heart Lung and Blood Vessel Diseases, Beijing Anzhen Hospital, Capital Medical University, Beijing 100029, China; State Key Laboratory of Organ Regeneration and Reconstruction, Institute of Zoology, Chinese Academy of Sciences, Beijing 100101, China; University of Chinese Academy of Sciences, Beijing 100049, China; Beijing Institute for Stem Cell and Regenerative Medicine, Beijing 100101, China; Aging Biomarker Consortium (ABC), Beijing 100101, China; University of Chinese Academy of Sciences, Beijing 100049, China; Aging Biomarker Consortium (ABC), Beijing 100101, China; China National Center for Bioinformation and Beijing Institute of Genomics, Chinese Academy of Sciences, Beijing 100101, China; Sino-Danish College, University of Chinese Academy of Sciences, Sino-Danish Center for Education and Research, Beijing 101408, China; State Key Laboratory of Organ Regeneration and Reconstruction, Institute of Zoology, Chinese Academy of Sciences, Beijing 100101, China; University of Chinese Academy of Sciences, Beijing 100049, China; Beijing Institute of Heart Lung and Blood Vessel Diseases, Beijing Anzhen Hospital, Capital Medical University, Beijing 100029, China; Beijing Key Laboratory of Environmental Toxicology, School of Public Health, Capital Medical University, Beijing 100069, China; Aging Biomarker Consortium (ABC), Beijing 100101, China; Center for Reproductive Medicine, Cheeloo College of Medicine, Shandong University, Jinan 250012, China; Key Laboratory of Reproductive Endocrinology of Ministry of Education, National Research Center for Assisted Reproductive Technology and Reproductive Genetics, Shandong Key Laboratory of Reproductive Medicine, Shandong Provincial Clinical Research Center for Reproductive Health, Jinan 250012, China; Aging Biomarker Consortium (ABC), Beijing 100101, China; Oujiang Laboratory, Center for Geriatric Medicine and Institute of Aging, Key Laboratory of Alzheimer’s Disease of Zhejiang Province, Zhejiang Provincial Clinical Research for Mental Disorders, The First-affiliated Hospital, Wenzhou Medical University, Wenzhou 325035, China; Aging Biomarker Consortium (ABC), Beijing 100101, China; Center for Stem Cell Biology and Tissue Engineering, Key Laboratory for Stem Cells and Tissue Engineering, Ministry of Education, Sun Yat-sen University, Guangzhou 510000, China; Department of Biochemistry, Zhongshan School of Medicine, Sun Yat-sen University, Guangzhou 510000, China; Altos Labs San Diego Institute of Science, San Diego, CA 94022, United States; Salk Institute for Biological Studies, La Jolla, CA 92037, USA; Advanced Innovation Center for Human Brain Protection, National Clinical Research Center for Geriatric Disorders, Aging Translational Medicine Center, Beijing Municipal Geriatric Medical Research Center, Beijing Key Laboratory of Environment and Aging, Xuanwu Hospital Capital Medical University, Beijing 100053, China; State Key Laboratory of Organ Regeneration and Reconstruction, Institute of Zoology, Chinese Academy of Sciences, Beijing 100101, China; University of Chinese Academy of Sciences, Beijing 100049, China; Beijing Institute for Stem Cell and Regenerative Medicine, Beijing 100101, China; Aging Biomarker Consortium (ABC), Beijing 100101, China; Human Organ Physiopathology Emulation System, Institute of Zoology, Chinese Academy of Sciences, Beijing 100101, China; State Key Laboratory of Organ Regeneration and Reconstruction, Institute of Zoology, Chinese Academy of Sciences, Beijing 100101, China; University of Chinese Academy of Sciences, Beijing 100049, China; Beijing Institute of Heart Lung and Blood Vessel Diseases, Beijing Anzhen Hospital, Capital Medical University, Beijing 100029, China; Beijing Institute for Stem Cell and Regenerative Medicine, Beijing 100101, China; Aging Biomarker Consortium (ABC), Beijing 100101, China; Human Organ Physiopathology Emulation System, Institute of Zoology, Chinese Academy of Sciences, Beijing 100101, China; Advanced Innovation Center for Human Brain Protection, National Clinical Research Center for Geriatric Disorders, Aging Translational Medicine Center, Beijing Municipal Geriatric Medical Research Center, Beijing Key Laboratory of Environment and Aging, Xuanwu Hospital Capital Medical University, Beijing 100053, China; Aging Biomarker Consortium (ABC), Beijing 100101, China

**Keywords:** primate, epididymis, aging, single-nucleus transcriptomics, longevity gene, FOXO1, cell therapy

## Abstract

Aging of the male reproductive system is characterized by declining fertility, with epididymal dysfunction being a critical yet poorly understood contributor. Through a multimodal analysis in non-human primates that integrated histology and transcriptomics, we delineated a coherent epididymal aging phenotype encompassing epithelial senescence, chronic inflammation, fibrosis, and functional decline. Single-nucleus transcriptomics revealed principal cells (PCs) as the predominant and most transcriptionally perturbed epithelial cell type. Within PCs, the longevity-associated transcription factor FOXO1 was markedly downregulated with age. Functional studies in human epididymal epithelial cells demonstrated that FOXO1 deficiency drives cellular senescence. Mechanistically, FOXO1 transcriptionally activates LHX1, and this axis is essential for counteracting senescence. Furthermore, intervention with senescence-resistant mesenchymal progenitor cells or their exosomes mitigated epididymal aging phenotypes and restored FOXO1 expression *in vivo* and *in vitro*. Our study establishes the FOXO1-LHX1 axis as a key protective pathway against primate epididymal aging, providing mechanistic insights and potential therapeutic targets for preserving male reproductive health.

## Introduction

Aging profoundly impacts reproductive physiology, leading to a well-documented decline in fertility and alterations in endocrine function in both sexes (Akhigbe et al., 2025; Gunes et al., 2016; Hermann et al., 2000; Huang et al., 2025; Jiang et al., 2024; Kaufman et al., 2019; Pan et al., 2025; Tang et al., 2025). While age-related degeneration of the testes is a primary contributor to late-onset hypogonadism and male infertility, emerging evidence underscores a significant yet underappreciated role for functional decline in the extra-testicular reproductive tract (Cai et al., 2022; Gunes et al., 2016; Matsumoto, 2002; Nieschlag, 2020; Pino et al., 2020). Among these, the epididymis is particularly crucial, as it is indispensable for post-testicular sperm maturation, storage, and the acquisition of fertilizing capacity (Cornwall, 2009; Sullivan and Mieusset, 2016). Its pseudostratified epithelium, composed notably of principal cells (PCs), creates a specialized luminal microenvironment through active secretion and the maintenance of the blood-epididymis barrier (Long et al., 2024; Vinay et al., 2025). Despite its critical function, the cellular and molecular mechanisms underlying epididymal aging remain poorly characterized. A key knowledge gap is the lack of a systematic, cell-type-resolved understanding of how the tissue remodels with age, which hinders the development of strategies to mitigate male reproductive aging.

Recent advances in single-cell RNA sequencing (scRNA-seq) offer an unprecedented opportunity to decode complex tissue dynamics, including those driving aging and pathology (Aging Biomarker Consortium et al., 2023; Jing et al., 2025; Lei et al., 2025; Mao et al., 2024; Wang et al., 2020; Yang et al., 2024; Zhao et al., 2025; Zhu et al., 2023). Although scRNA-seq has been applied to characterize cellular heterogeneity in the epididymis and its response to metabolic stress (Rinaldi et al., 2020; Sárvári et al., 2021; Shi et al., 2021; Yan et al., 2024; Zhang et al., 2024), a comprehensive atlas detailing cell-type-specific transcriptional changes during physiological aging in primates is absent. Such a study is ethically and practically challenging in humans due to limited access to healthy, age-matched tissue samples. Non-human primates (NHPs), with their close phylogenetic relationship and high physiological fidelity to humans in reproductive anatomy, endocrinology, and immunology (Sun et al., 2026; van Der Horst et al., 1999; Wang et al., 2020), provide a powerful and translationally relevant model system for investigating the fundamental biology of reproductive aging.

In this study, we performed a multi-omics investigation of epididymal aging in the crab-eating macaque (*Macaca fascicularis*). We combined histological analysis, bulk RNA sequencing, and single-nucleus transcriptomics to construct a high-resolution phenotypic and molecular map of the aging primate epididymis. Our analysis identified cellular senescence, chronic inflammation, and fibrosis as hallmarks of epididymal aging. At the single-cell level, we discovered that PCs undergo the most pronounced transcriptional disturbance. We further identified and functionally validated the downregulation of the transcription factor FOXO1 as a key driver of PC senescence. Mechanistically, we delineate a novel pathway in which FOXO1 transcriptionally activates *LHX1* to exert its geroprotective effects. Finally, we demonstrate that intervention with senescence-resistant mesenchymal progenitor cells (SRCs) or their derived exosomes can ameliorate key aging phenotypes. This work provides a foundational cellular and molecular framework for understanding epididymal aging, revealing new targets for potential interventions aimed at preserving male reproductive health.

## Results

### Characterization of aging-related phenotypes in the NHP epididymis

**Figure 1.**
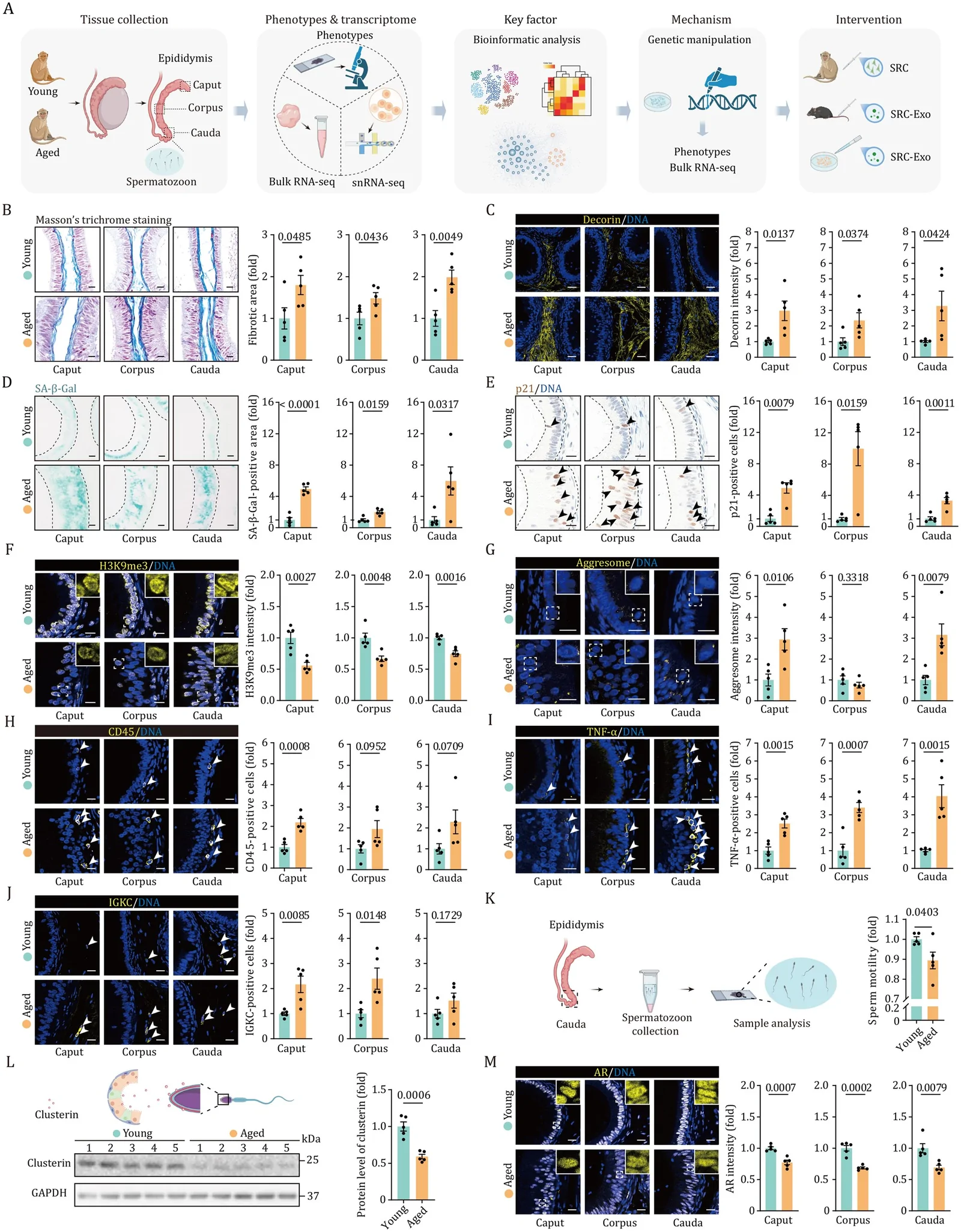
**Characterization of aging-related phenotypes in cynomolgus monkey epididymis**. (A) Overview of the integrated experimental workflow, including histological analysis, bulk RNA sequencing (RNA-seq), single-nucleus RNA sequencing (snRNA-seq), and subsequent mechanistic investigation. Schematic diagrams were generated using BioRender.com. (B) Representative Masson’s trichrome staining images in the caput, corpus, and cauda epididymis of young and aged monkeys. Collagen fibers are stained blue by aniline blue. Collagen fibers-positive area was quantified as fold changes (aged vs. young). Scale bars, 20 μm. *n *= 5 monkeys for each group. (C) Decorin immunostaining in the caput, corpus, and cauda epididymis of young and aged monkeys. The relative intensity was quantified as fold changes (aged vs. young). Scale bars, 20 μm. *n *= 5 monkeys for each group. (D) SA-β-Gal staining in the caput, corpus, and cauda epididymis of young and aged monkeys. SA-β-Gal-positive area in the indicated region was quantified as fold changes (aged vs. young). Scale bars, 20 μm. *n *= 5 monkeys for each group. (E) p21 immunohistochemical staining in the caput, corpus, and cauda epididymis of young and aged monkeys. The numbers of p21-positive cells were quantified as fold changes (aged vs. young). The arrowheads indicate p21-positive cells. Scale bars, 20 μm. *n *= 5 monkeys for each group. (F) H3K9me3 immunostaining in the caput, corpus, and cauda epididymis of young and aged monkeys. The relative intensity of H3K9me3 in the epididymal epithelium was quantified as fold changes (aged vs. young). Scale bars, 20 μm. *n *= 5 monkeys for each group. (G) Aggresome staining in the caput, corpus, and cauda epididymis of young and aged monkeys. The relative intensity of aggresome in epididymal epithelium was quantified as fold changes (aged vs. young). Scale bars, 20 μm. *n *= 5 monkeys for each group. Data are presented as means ± SEM. (H) CD45 immunostaining in the caput, corpus, and cauda epididymis of young and aged monkeys. The numbers of CD45-positive cells were quantified as fold changes (aged vs. young). The arrowheads indicate CD45-positive cells. Scale bars, 20 μm. *n *= 5 monkeys for each group. (I) TNF-α immunostaining in the caput, corpus, and cauda epididymis of young and aged monkeys. The numbers of TNF-α-positive cells were quantified as fold changes (aged vs. young). The arrowheads indicate TNF-α-positive cells. Scale bars, 20 μm. *n *= 5 monkeys for each group. (J) IGKC immunostaining in the caput, corpus, and cauda epididymis of young and aged monkeys. The numbers of IGKC-positive cells were quantified as fold changes (aged vs. young). The arrowheads indicate IGKC-positive cells. Scale bars, 20 μm. *n *= 5 monkeys for each group. (K) Assessment of sperm motility in the cauda epididymis of monkeys. *n *= 5 monkeys for each group. (L) Western blot analysis of clusterin protein levels in the epididymis of young and aged monkeys. The α-subunit of clusterin was detected. GAPDH is used as a loading control. The relative protein expression levels were quantified as fold changes (aged vs. young). *n *= 5 monkeys for each group. (M) Androgen Receptor (AR) immunostaining in the caput, corpus, and cauda epididymis of young and aged monkeys. The relative intensity of AR in epididymal epithelium was quantified as fold changes (aged vs. young). Scale bars, 20 μm. *n *= 5 monkeys for each group. For B-M, data are presented as means ± SEM. Two-tailed Student’s *t*-test (for normally distributed data) or two-tailed Wilcoxon rank-sum test (for non-normally distributed data) are used.

To systematically define the phenotypic landscape of epididymal aging, we employed a non-human primate (NHP) model, the crab-eating macaque (*Macaca fascicularis*), which recapitulates key aspects of human reproductive physiology (Jing et al., 2025; Lu et al., 2024; Wang et al., 2020). We conducted a comparative analysis using five young adults (5–6 years old; analogous to humans 18–20 years old) and five aged (16–18 years old; ∼60 human years) individuals (Fig. 1A).

Histopathological assessment revealed pronounced structural alterations in aged epididymides. Masson’s trichrome staining and decorin immunolabeling demonstrated a marked increase in interstitial collagen deposition and fibrosis across three major anatomical regions (caput, corpus, and cauda) in aged animals (Fig. 1B and 1C). This was accompanied by a widespread accumulation of cells positive for senescence markers, including senescence-associated β-galactosidase (SA-β-Gal) and the cyclin-dependent kinase inhibitor p21 (Fig. 1D and 1E). At the subcellular level, we observed a reduction in the heterochromatin marker H3K9me3 and an accumulation of protein aggregates (aggresomes), indicating concomitant loss of epigenetic stability and proteostasis (Fig. 1F and 1G). The aged epididymal microenvironment exhibited hallmarks of chronic inflammation. Immunostaining showed increased infiltration of CD45^+^ leukocytes and CD68^+^ macrophages (Figs. 1H and S1A). Consistent with an activated senescence-associated secretory phenotype (SASP), expression of the pro-inflammatory cytokine TNF-α was elevated (Fig. 1I). Furthermore, levels of IGKC—an immunoglobulin light chain recently implicated as a biomarker of tissue aging (Ma et al., 2024; Walters, 2024)—were substantially higher in aged tissues (Fig. 1J).

Functional correlates of these structural changes were evident. Sperm motility in the cauda epididymis, a direct readout of luminal microenvironment quality, was impaired in aged monkeys (Fig. 1K). Molecular analysis identified two key alterations linked to epithelial function: first, a substantial decrease in the secretion of clusterin, a PC-derived glycoprotein vital for sperm acrosomal function (Saewu et al., 2017) (Fig. 1L); and second, a marked reduction in androgen receptor (AR) expression within the epididymal epithelium of aged individuals (Fig. 1M). This diminished AR signaling was associated with a decline in Ki67-positive proliferating epithelial cells (Fig. S1B), aligning with the established role of androgen signaling in maintaining epithelial homeostasis (Hamzeh and Robaire, 2009; Kim and Breton, 2020; O’Hara et al., 2011; Yang et al., 2018).

In summary, our multimodal analysis delineates a coherent phenotype of primate epididymal aging, encompassing tissue fibrosis, epithelial senescence, inflammatory microenvironment remodeling, and functional decline, thereby providing a histological and functional foundation for subsequent mechanistic investigation.

### Global transcriptional profiling of non-human primate epididymal aging

**Figure 2.**
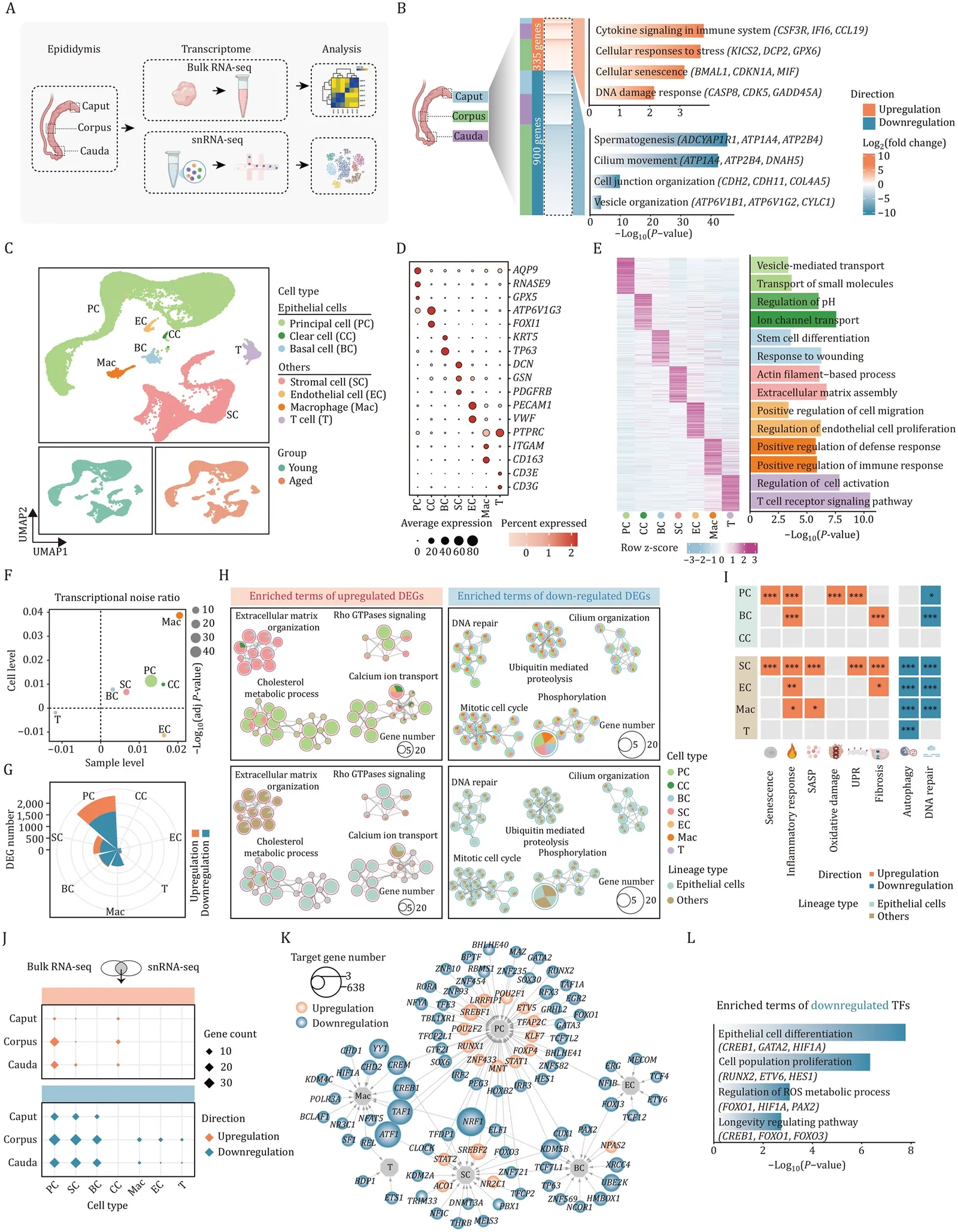
**Global transcriptional profiling of epididymal aging in non-human primates**. (A) Workflow diagram of bulk RNA-seq and snRNA-seq of three anatomical regions (caput, corpus, and cauda) of the epididymis, along with corresponding data analysis. (B) Age-associated transcriptional changes in the monkey epididymis. The left heatmap shows Log_2_(fold change) of differentially expressed genes (DEGs) between aged and young groups across the three anatomical regions of the epididymis based on bulk RNA-seq. The right panel highlights biological pathways enriched among up- and down-regulated DEGs. (C) Uniform manifold approximation and projection (UMAP) plot shows the distribution of different cell types in epididymis. (D) Dot plot shows the expression of representative marker genes for each cell type in the epididymis. (E) Heatmap depicts the expression profiles of top 50 marker genes across different cell types in young and aged monkey epididymis, with enriched functional annotations shown on the right. (F) Scatterplot shows the log_2_ ratio of transcriptional noise between aged and young samples as calculated using monkey averages and single cells on the X and Y axes, respectively. (G) Circular bar plot shows the number of up- and down-regulated genes from epididymal snRNA-seq data. (H) Network represents enriched terms and pathways of aging DEGs across different cell types in the monkey epididymis. The upper panel is organized by cell type, while the lower panel is organized by lineage type. The nodes represent enriched terms. The pie charts show the proportion of number of genes that hit the certain term across cell types. (I) Heatmap shows changes in genes related to senescence, inflammatory response, SASP, oxidative damage, UPR, fibrosis, autophagy, and DNA repair in young and aged monkey epididymis tissues, based on AUC scores. Statistical significance was assessed using the Wilcoxon rank-sum test with FDR correction: *P < 0.05, **P < 0.01, ***P < 0.001. (J) Integrated analysis of DEGs from bulk RNA-seq and snRNA-seq data of the monkey epididymis. (K) Network plot shows up- and down-regulated core transcription factors (TFs) across all cell types during epididymis aging. The circular nodes represent core TFs and the hexagonal nodes indicate cell types, the node size of TF indicates the number of target gene hits, and the node color of TF indicates whether they are upregulated or downregulated with aging. (L) Bar plot illustrates representative enriched terms of downregulated TFs.

To delineate the genome-wide molecular alterations underlying epididymal aging, we performed bulk RNA sequencing (RNA-seq) on tissue samples from the three principal anatomical regions—caput, corpus, and cauda—of young and aged crab-eating macaques (Figs. 2A, S2A and S2B). Comparative transcriptomic analysis across these regions between age groups identified a total of 1,235 aging-associated differentially expressed genes (DEGs), with 335 genes upregulated and 900 genes downregulated in aged animals (Fig. 2B; Table S1).

Functional enrichment analysis of age-related DEGs revealed programmatic molecular shifts underlying primate epididymal aging. Upregulated genes were enriched in pathways related to senescence and cytokine signaling (Fig. 2B), correlating with histological hallmarks of increased immune infiltration and elevated TNF-α (Figs. 1H, 1I, and S1A). Conversely, downregulated genes mapped to processes critical for epithelial integrity and secretion—including cell-cell junctions, cilium function, and vesicle transport (Fig. 2B)—a finding that provided a molecular basis for the observed decline in secretion of key proteins like clusterin (Fig. 1L) and suggested a broad impairment of the blood-epididymis barrier and luminal microenvironment (James et al., 2020; Li et al., 2025a; Zhou et al., 2018). Thus, our bulk transcriptomic analysis links tissue degeneration to specific dysregulation of inflammatory signaling and epithelial functional pathways, underscoring that epididymal aging is characterized by a pervasive inflammatory response coupled with a functional recession.

### Cell taxonomy of non-human primate epididymal aging

To resolve the cell-type-specific gene expression dynamics of epididymal aging, we established a single-nucleus transcriptomic atlas of the aging primate epididymis (Fig. 2C). After rigorous quality control and filtering, we obtained 90,715 high-quality single-nucleus transcriptomes for downstream analysis (Fig. S2C and S2D). Uniform manifold approximation and projection (UMAP) visualization segregated these transcriptomes into distinct clusters, representing the major cellular constituents of the tissue (Fig. 2C).

Using established canonical marker genes, we annotated seven major cell types: principal cell (PC; expressing *AQP9*, *RNASE9*, *GPX5*, *CRISP1*; 65.12%), clear cell (CC; *ATP6V1G3*, *FOXI1*; 0.33%), basal cell (BC; *KRT5*, *TP63*; 2.35%), stromal cell (SC; *DCN*, *GSN*, *PDGFRB*; 26.37%), endothelial cell (EC; *PECAM1*, *VWF*; 1.01%), macrophage (Mac; *PTPRC*, *ITGAM*, *CD163*; 1.90%), and T cell (T; *CD3E*, *CD3G*; 2.92%) (Figs. 2D, 2E, and S2E; Table S2). Consistent with their known role as the primary functional epithelium, PCs constituted the largest cellular compartment (James et al., 2020; Leir et al., 2020) (Fig. S2F). Functional enrichment of the top 50 marker genes for each type confirmed physiologically relevant signatures: PC markers were linked to vesicle and small molecule transport; BC markers were associated with stem cell differentiation pathway, aligning with their role in epithelial maintenance; and immune-related pathways were concentrated in macrophage and T cell clusters (Fig. 2E).

Interrogation of this atlas revealed pervasive age-related alterations in cellular identity. A widespread decrease in cell-type-specific marker gene set scores across multiple lineages indicated a drift from canonical cellular states in aged animals (Fig. S2G), a phenomenon recognized as a hallmark of tissue aging (Connolly et al., 2024; Ma et al., 2024; Yang et al., 2023). Given that increased transcriptional noise can erode clear cell identity, we quantified this noise across cell types (Angelidis et al., 2019; Martinez-Jimenez et al., 2017). This analysis showed an elevation of transcriptional noise in most cell types from aged epididymides, with PCs exhibiting the most pronounced increase (Fig. 2F).

### Cell type-specific transcriptional dynamics during primate epididymal aging

To elucidate aging-associated transcriptomic alterations with cellular resolution, we performed comparative single-nucleus analysis between young and aged epididymides, identifying cell-type-specific DEGs (Fig. 2G; Table S1). Among epithelial and stromal populations, PCs, SCs, and BCs harbored the most extensive transcriptional changes with age (Fig. 2G). PCs showed 671 upregulated and 1,650 downregulated DEGs; SCs had 283 upregulated and 758 downregulated; and BCs exhibited 30 upregulated and 813 downregulated DEGs (Fig. 2G). Functional enrichment of these DEGs revealed coherent age-related alterations. Upregulated genes in aged cells were prominently associated with extracellular matrix organization, aligning with the histologically observed increase in tissue fibrosis (Fig. 2H). Notably, genes involved in calcium ion homeostasis were also upregulated, which may perturb the precisely regulated, low-calcium luminal microenvironment essential for normal sperm maturation and storage (Da Silva et al., 2007; Shum et al., 2022; Weissgerber et al., 2012). Conversely, downregulated DEGs were enriched in fundamental biological processes critical for tissue homeostasis, including cell cycle regulation (mitosis), signal transduction (phosphorylation), genomic maintenance (DNA repair), protein quality control (ubiquitin-mediated proteolysis), and epithelial structure (cilium organization) (Fig. 2H).

We next evaluated the expression of a curated panel of classical aging hallmark genes across cell types (López-Otín et al., 2013; Lu et al., 2024; Wu et al., 2025) (Table S3). PCs and SCs displayed the most pronounced alterations across pathways encompassing cellular senescence, inflammation, oxidative stress, and autophagy (Fig. 2I). Integration of bulk and single-nucleus data further underscored the central role of PCs, as this cell type contributed the largest number of genes to the overall aging transcriptomic signature (Fig. 2J), indicating that PCs undergo the most substantial transcriptional reprogramming during epididymal aging.

To uncover upstream regulators of these changes, we constructed a transcription factor (TF) regulatory network. This analysis identified several aging-dysregulated TFs: pro-inflammatory factors such as STAT1 and STAT2 were upregulated, while TFs involved in stress response and longevity, including HIF1A, FOXO1, and FOXO3, were downregulated in aged tissues (Alazawi et al., 2013; Carelock et al., 2023; Geng et al., 2023; Hwang and Lee, 2011; Li et al., 2025b; Martins et al., 2016; Zhao et al., 2022; Zheng et al., 2025) (Fig. 2K and 2L). Cell–cell communication analysis revealed a general attenuation of interaction across the aged epididymis, with specific alterations in signaling networks. Communication among PCs, BCs, ECs, and macrophages was weakened, whereas interactions involving CCs were relatively strengthened (Fig. S2H). The upregulation of the chemokine CCL11 (eotaxin-1) and its scavenger receptor ACKR4 suggests a dysregulated and potentially compensatory immune microenvironment in aged tissue (Ivanovska et al., 2020; Naser et al., 2024).

Finally, we contextualized our DEGs within broader aging and disease frameworks. Cross-referencing with the Aging Atlas database confirmed the downregulation of conserved aging/longevity-related genes like *FOXO1*, *FOXO3*, and *CLOCK* in the aged epididymis (Aging Atlas Consortium et al., 2021; Chen et al., 2024; Wang et al., 2025) (Fig. S2I). Integration with pathogenic gene sets from the Harmonizome database (Diamant et al., 2025) revealed that DEGs from PCs showed the greatest overlap with genes associated with various epididymal pathologies, including epididymitis and epithelial degeneration (Fig. S2J). For example, *MFGE8*, which was upregulated in aged PCs and linked to inflammatory responses (Das et al., 2016; Deroide et al., 2013; Last et al., 2020), was also associated with epididymitis, suggesting a direct connection between the aging transcriptome and disease susceptibility.

### Age-related molecular alterations in the aged primate epididymal epithelium

**Figure 3.**
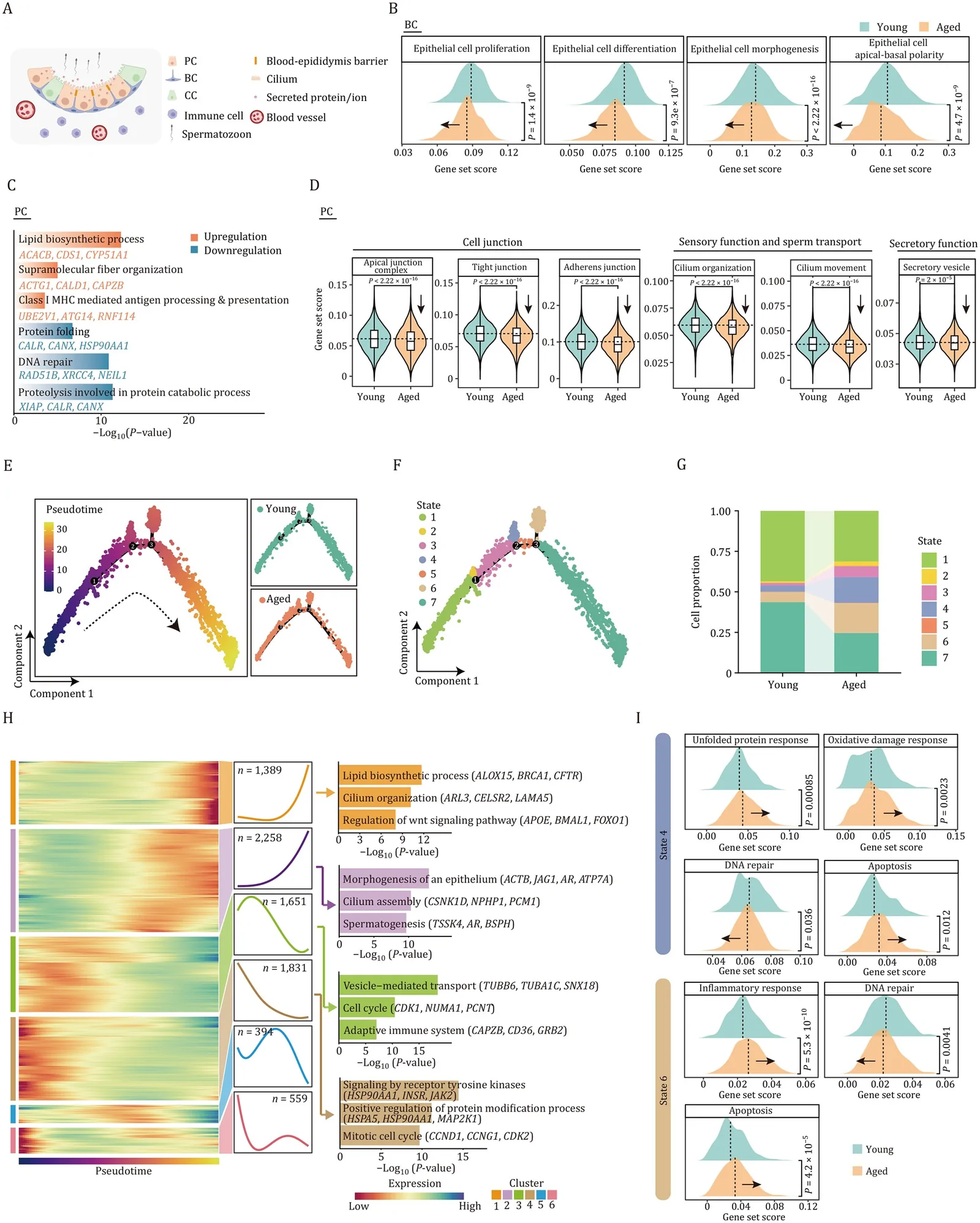
**Transcriptomic alterations associated with aging in non-human primate epididymal epithelial cells**. (A) Schematic representation of the structure of the epididymal epithelium. (B) AUC scores for gene sets associated with epithelial cell proliferation, differentiation, morphogenesis, and apical-basal polarity in basal cells. Two-tailed Wilcoxon rank-sum tests are performed. (C) Bar plot shows enriched terms of aging DEGs in principal cells (PCs) of the epididymis from aged and young monkeys. (D) AUC scores of gene sets related to epididymal epithelial structure and function in principal cells. The dot plot below represents the scaled median scores. Two-tailed Wilcoxon rank-sum tests are performed. (E) Pseudotime analysis of PCs in NHP epididymis. Left, pseudotime scores of PCs. Right, the distribution of young and aged groups along the pseudotime trajectory. (F) The distribution of different states of PCs along the pseudotime trajectory. (G) Stacked bar plot shows the proportions of different PC states in the aged and young groups. (H) Heatmaps, curve charts, and bar plots show the expression profiles of the genes that change along the PC pseudotime trajectory. According to the expression pattern, DEGs are divided into six clusters. Left, heatmap shows the expression levels of the DEGs. Right, curve charts and bar plots shows the expression patterns and enriched terms of the corresponding pattern. (I) Ridge plots show the gene set scores of the aging-related gene sets indicated in the figure for PCs in state 4 and state 6. The black arrow indicates the direction of median score shift in the aged group compared to young group. Two-tailed Wilcoxon rank-sum tests were performed.

The epididymal epithelium, as the primary functional unit of the organ, is central to the structural and functional decline observed with advancing age (Cornwall, 2009) (Fig. 3A). We observed pronounced functional regression in two key epithelial populations. BCs, which are postulated to serve as epithelial progenitors (Pinel et al., 2019), exhibited a marked reduction in proliferative and differentiation potential, accompanied by a loss of apical–basal polarity in aged individuals (Fig. 3B; Table S3). PC, the most abundant epithelial type and the one displaying the greatest transcriptional sensitivity to age, manifested a broad spectrum of degenerative changes. These included increased intracellular lipid accumulation, an enhanced inflammatory gene signature, and a coordinated downregulation of pathways vital for cellular homeostasis, such as protein folding and degradation, and DNA repair (Fig. 3C). To directly link transcriptional changes to functional regression, we assessed gene sets governing epithelial integrity and secretion. In aged PCs, we observed a downregulation of genes encoding core components of the apical junctional complex, including critical elements of tight junctions and adherens junctions (Fig. 3D). This transcriptional decline suggests a molecular basis for the potential compromise of the blood-epididymis barrier (Gregory and Cyr, 2014; Zhao et al., 2020). Concurrently, gene set scores associated with ciliogenesis and ciliary function, as well as vesicle-mediated secretion, were substantially lower in aged PCs (Fig. 3D). Together, these findings indicate a multidimensional functional decline in the aging epithelium, encompassing both its structural barrier properties, and its specialized secretory capacity.

To map the continuum of PC states during aging, we reconstructed a unified pseudotemporal trajectory integrating cells from young and aged epididymides. This analysis resolved seven distinct transcriptional states (Fig. 3E–G). Gene expression dynamics along the trajectory revealed a progressive downregulation of genes involved in cell cycle progression and vesicle-mediated transport, in contrast to a sustained increase in expression of gene modules dedicated to ciliary organization, lipid metabolism, and sperm maturation support (Fig. 3H). Notably, the proportion of PCs occupying specific states shifted with age. States 4 and 6, which were enriched in aged samples (Fig. 3G), were characterized by transcriptional signatures indicative of elevated reactive oxygen species, heightened immune activation, and increased apoptotic priming (Fig. 3I). This shift in cellular composition highlights a functional skewing of the aged epithelial population toward pro-inflammatory and stress-associated phenotypes.

### FOXO1 serves as a geroprotective transcription factor in primate epididymal aging

**Figure 4.**
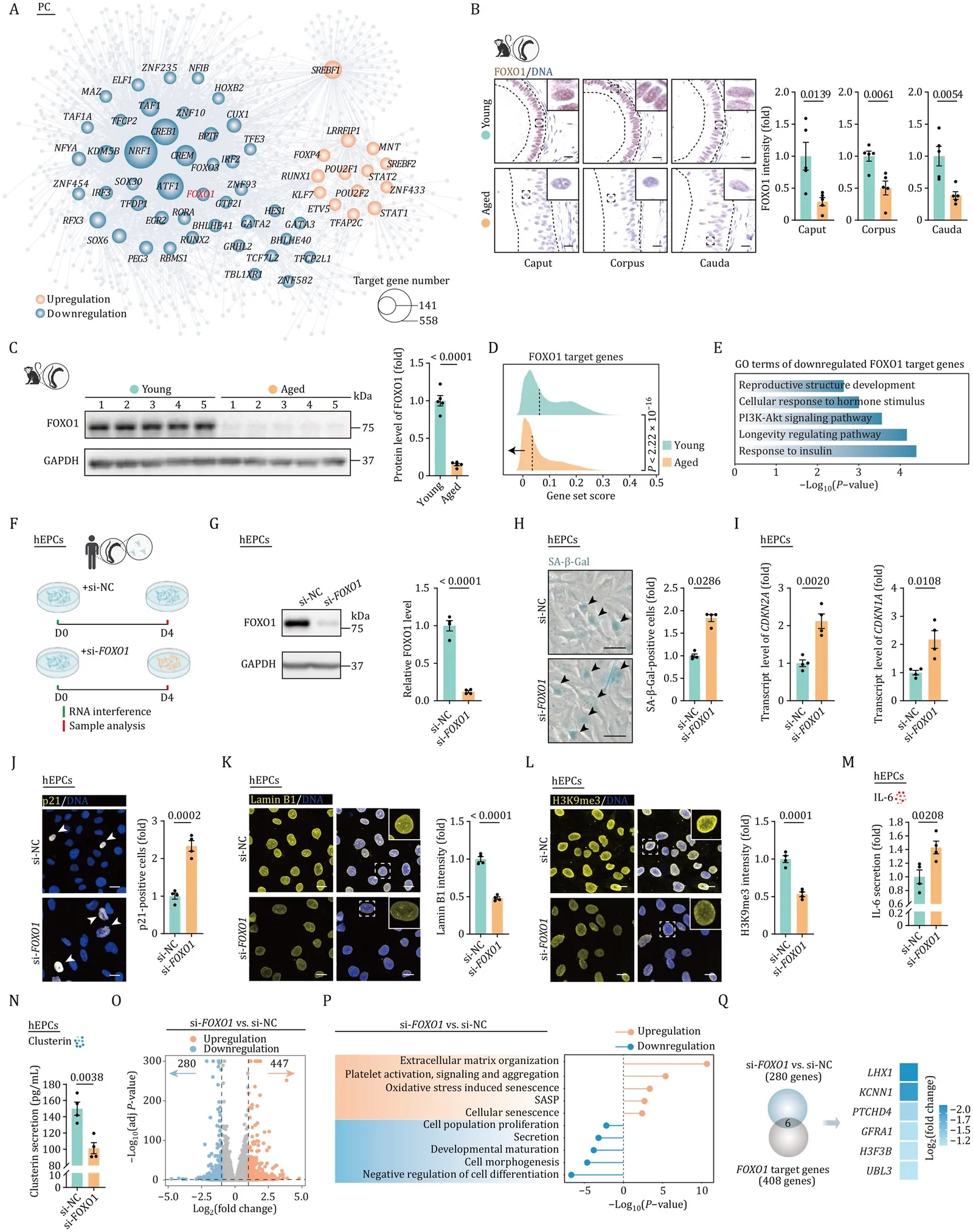
**Depletion of FOXO1 accelerated cellular senescence in human epididymal principal cells**. (A) Network plot shows up- and down-regulated core TFs of PCs during epididymal aging. Node size represents the number of target genes for each TF, and the node color of TF indicates whether the TF is up- or down-regulated with aging. (B) Immunohistochemical staining of FOXO1 in the caput, corpus, and cauda epididymis of young and aged monkeys. The relative intensity of FOXO1 in epididymal epithelium was quantified as fold changes (aged vs. young). Scale bars, 20 μm. Young, *n *= 5; Aged, *n *= 5. (C) Western blot analysis of FOXO1 levels in epididymal tissues from young and aged monkeys. GAPDH is used as the loading control. Young, *n *= 5; Aged, *n *= 5. (D) Ridge map shows the global distribution density of AUC score of FOXO1 target genes in PC. The corresponding dashed lines represent the median of each group. A two-tailed Wilcoxon rank-sum test was performed. (E) Bar plot shows representative enriched terms of downregulated FOXO1 target genes. (F) The schematic illustrates the culture and treatment scheme for human epididymal principal cells (hEPCs) subjected to si-NC or si-*FOXO1* transfection, with detailed experimental conditions provided in the figure. (G) Western blot analysis of FOXO1 levels in hEPCs following transfection with si-NC or si-*FOXO1*. *n* = 4 for each group. (H) Assessment of SA-β-Gal positivity in hEPCs following transfection with si-NC or si-*FOXO1*. The arrowheads indicate SA-β-Gal-positive hEPCs. Scale bars, 20 μm. *n* = 4 for each group. (I) RT-qPCR analysis for mRNA levels of *CDKN2A* and *CDKN1A* in hEPCs following transfection with si-NC or si-*FOXO1*. *n* = 4 for each group. (J) Immunofluorescence analysis of p21 in hEPCs following transfection with si-NC or si-*FOXO1*. The arrowheads indicate p21-positive hEPCs. Scale bars, 20 μm. *n* = 4 for each group. (K) Immunofluorescence examination of Lamin B1 in hEPCs following transfection with si-NC or si-*FOXO1*. Scale bars, 20 μm. *n* = 4 for each group. (L) Immunofluorescence examination of H3K9me3 in hEPCs following transfection with si-NC or si-*FOXO1*. Scale bars, 20 μm. *n* = 4 for each group. (M) The secretion level of IL-6 in the culture supernatant, collected from 1 × 10^5^ hEPCs treated with si-NC or si-*FOXO1*, was measured by ELISA, with the si-NC group serving as the control. *n* = 4 for each group. (N) The secretion level of clusterin in the culture supernatant, collected from 1 × 10^5^ hEPCs treated with si-NC or si-*FOXO1*, was measured by ELISA, with the si-NC group serving as the control. *n* = 4 for each group. (O) Volcano plot shows DEGs in hEPCs following transfection with si-NC or si-*FOXO1*. (P) Lollipop plot illustrates representative pathways enriched in DEGs of hEPCs following transfection with si-NC or si-*FOXO1*. (Q) Venn diagram illustrates the overlap between downregulated genes following *FOXO1* knockdown in hEPCs and FOXO1 target genes curated from the TRANSFAC and ENCODE databases. The accompanying heatmap shows Log_2_(fold change) of the overlapping genes. For (B–C and G–N), data are presented as means ± SEM. Two-tailed Student’s *t*-test (for normally distributed data) and two-tailed Wilcoxon rank-sum test (for non-normally distributed data) are used.

To identify upstream regulators driving PC aging, we analyzed TFs governing the age-related DEGs in PCs. This analysis pinpointed FOXO1 as a core TF whose expression was downregulated in aged PCs (Fig. 4A). Immunohisto­chemical staining and Western blot analysis confirmed a pronounced reduction of FOXO1 protein in the epididymis of aged cynomolgus monkeys (Fig. 4B and 4C). Interrogation of the FOXO1 regulatory network revealed a broad downregulation of its predicted target genes in aged PCs (Fig. 4D). Functional enrichment of these downregulated targets highlighted pathways related to longevity and reproductive system development (Fig. 4E), implicating FOXO1 dysfunction in the degenerative processes associated with aging.

We next established a direct causal link between FOXO1 loss and cellular senescence using primary human epididymal PCs (hEPCs), which were validated by marker expression and clusterin secretory function (Fig. S3A and S3B). siRNA-mediated knockdown of *FOXO1* precipitated a robust senescent phenotype (Fig. 4F and 4G). This was evidenced by an increase in SA-β-Gal activity (Fig. 4H), elevated levels of *CDKN2A* (p16) and *CDKN1A* (p21) (Fig. 4I and 4J), loss of the nuclear lamina component Lamin B1 and the heterochromatin marker H3K9me3 (Fig. 4K and 4L), and increased secretion of the SASP factor IL-6 (Fig. 4M). The cells’ specialized secretory function was concurrently impaired, as shown by reduced clusterin secretion (Fig. 4N). Bulk RNA-seq of FOXO1-deficient hEPCs corroborated these findings, demonstrating upregulated senescence/SASP pathways and downregulated epithelial secretion programs (Fig. 4O and 4P; Table S4).

### FOXO1 counteracts cellular senescence by transactivating *LHX1*

**Figure 5.**
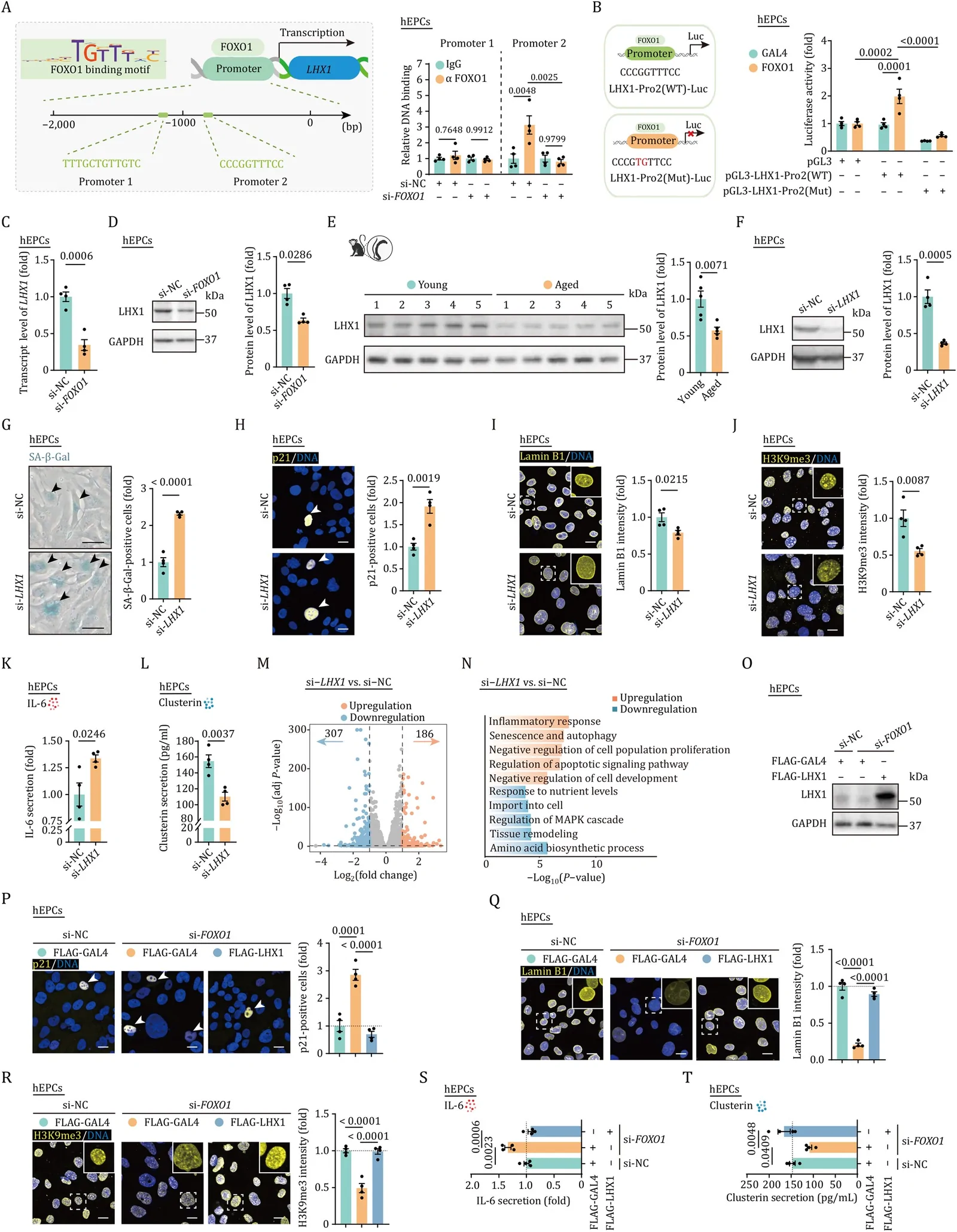
**FOXO1 counteracts human epididymal principal cell senescence by transactivating LHX1**. (A) The enrichment of the *LHX1* promoter in hEPCs treated with si-NC or si-*FOXO1* is assessed by ChIP-qPCR. The FOXO1 binding motif is derived from HOCOMOCO. *n* = 4 for each group. (B) Relative luciferase activity in cells co-transfected with pGL3-Promoter (pGL3), pGL3-LHX1-Promoter2 (pGL3-LHX1-Pro2 (WT)), and pGL3-LHX1-Promoter2 (mutation) (pGL3-LHX1-Pro2 (Mut))-driven firefly luciferase construct and Renilla luciferase control, transduced with lentiviruses expressing FLAG-GAL4 or FLAG-FOXO1. *n* = 4 for each group. (C) Transcript levels of *LHX1* in hEPCs following transfection with si-NC or si-*FOXO1*. *n* = 4 for each group. (D) Western blot analysis of LHX1 levels in hEPCs following transfection with si-NC or si-*FOXO1*. *n* = 4 for each group. (E) Western blot analysis of LHX1 levels in epididymal tissues from young and aged monkeys. *n* = 5 monkeys for each group. GAPDH is used as the loading control. *n *= 5 monkeys for each group. (F) Western blot analysis showing the protein levels of LHX1 in si-NC and si-*LHX1* hEPCs. GAPDH is used as the loading control. *n* = 4 for each group. (G) Assessment of SA-β-Gal positivity in hEPCs following transfection with si-NC or si-*LHX1*. The arrowheads indicate SA-β-Gal-positive hEPCs. Scale bars, 20 μm. *n* = 4 for each group. (H) Immunofluorescence analysis of p21 in hEPCs following transfection with si-NC or si-*LHX1*. The arrowheads indicate p21-positive hEPCs. Scale bars, 20 μm. *n* = 4 for each group. (I) Immunofluorescence examination of Lamin B1 in hEPCs following transfection with si-NC or si-*LHX1*. Scale bars, 20 μm. *n* = 4 for each group. (J) Immunofluorescence examination of H3K9me3 in hEPCs following transfection with si-NC or si-*LHX1*. Scale bars, 20 μm. *n* = 4 for each group. (K) The secretion level of IL-6 in the culture supernatant, collected from 1 × 10^5^ hEPCs treated with si-NC or si-*LHX1*, was measured by ELISA, with the si-NC group serving as the control. *n* = 4 for each group. (L) The secretion level of clusterin in the culture supernatant, collected from 1 × 10^5^ hEPCs treated with si-NC or si-*LHX1*, was measured by ELISA, with the si-NC group serving as the control. *n* = 4 for each group. (M) Volcano plot illustrates DEGs in hEPCs following transfection with si-NC or si-*LHX1*. (N) Bar plot illustrates representative pathways enriched in DEGs of hEPCs after transfection with si-NC or si-*LHX1*. (O) Western blot analysis of LHX1 protein levels in si-NC or si-*FOXO1* hEPCs transduced with lentiviruses expressing FLAG-GAL4 or FLAG-LHX1. (P) Immunofluorescence analysis of p21 in si-NC or si-F*OXO1* hEPCs transduced with lentiviruses expressing FLAG-GAL4 or FLAG-LHX1. The arrowheads indicate p21-positive hEPCs. Scale bars, 20 μm. *n* = 4 for each group. (Q) Immunofluorescence examination of Lamin B1 in si-NC or si-*FOXO1* hEPCs transduced with lentiviruses expressing FLAG-GAL4 or FLAG-LHX1. Scale bars, 20 μm. *n* = 4 for each group. (R) Immunofluorescence examination of H3K9me3 in si-NC or si-*FOXO1* hEPCs transduced with lentiviruses expressing FLAG-GAL4 or FLAG-LHX1. Scale bars, 20 μm. *n* = 4 for each group. (S) The secretion level of IL-6 in the culture supernatant, collected from 1 × 10^5^ hEPCs treated with si-NC or si-*FOXO1* and transduced with lentiviruses expressing FLAG-GAL4 or FLAG-LHX1, was measured by ELISA, with the si-NC group serving as the control. *n* = 4 for each group. (T) The secretion level of clusterin in the culture supernatant, collected from 1 × 10^5^ hEPCs treated with si-NC or si-*FOXO1* and transduced with lentiviruses expressing FLAG-GAL4 or FLAG-LHX1, was measured by ELISA, with the si-NC group serving as the control. *n* = 4 for each group. For C-L, data are presented as means ± SEM. Two-tailed Student’s *t*-test (for normally distributed data) and two-tailed Wilcoxon rank-sum test (for non-normally distributed data) are used. For (A, B and P–T), data are presented as means ± SEM, one-way ANOVA with Tukey’s multiple comparisons test *P* values are indicated.

To delineate the mechanistic pathway downstream of FOXO1, we intersected genes downregulated upon *FOXO1* knockdown with predicted FOXO1 target genes. *LHX1* emerged as the most substantially downregulated overlapping candidate (Fig. 4Q). Chromatin immunoprecipitation followed by qPCR (ChIP-qPCR) confirmed the direct binding of FOXO1 to the *LHX1* promoter (Fig. 5A). A dual-luciferase reporter assay further demonstrated that FOXO1 activates the *LHX1* promoter, and this transactivation was abrogated by mutation of the FOXO1 binding site (Fig. 5B). Consistent with this regulatory relationship, *FOXO1* knockdown reduced both *LHX1* mRNA and protein levels in hEPCs (Fig. 5C and 5D), while FOXO1 overexpression had the opposite effect (Fig. S3C and S3D). Moreover, LHX1 protein expression exhibited an age-dependent decline in cynomolgus monkey epididymis (Fig. 5E), mirroring the aging-related loss of FOXO1.

Functional studies established LHX1 as a critical downstream effector. Knockdown of *LHX1* in hEPCs recapitulated the senescence phenotypes observed with FOXO1 deficiency, including increased SA-β-Gal positivity, elevated p21 expression, loss of Lamin B1 and H3K9me3, elevated IL-6 secretion, and impaired clusterin production (Figs. 5F–L and S3E). Transcriptomic analysis confirmed the upregulation of senescence and inflammatory genes upon LHX1 loss (Fig. 5M and 5N; Table S4). Crucially, overexpression of LHX1 effectively rescued the senescence phenotypes induced by *FOXO1* knockdown. This was evidenced by reduced SA-β-Gal and p21 positivity, restored nuclear lamina and heterochromatin integrity, lowered IL-6 levels, and increased clusterin secretion (Figs. 5O–T and S3F–G). These results demonstrate that LHX1 acts as a key mediator of FOXO1’s geroprotective function in hEPCs.

### SRC intervention alleviates epididymal aging

**Figure 6.**
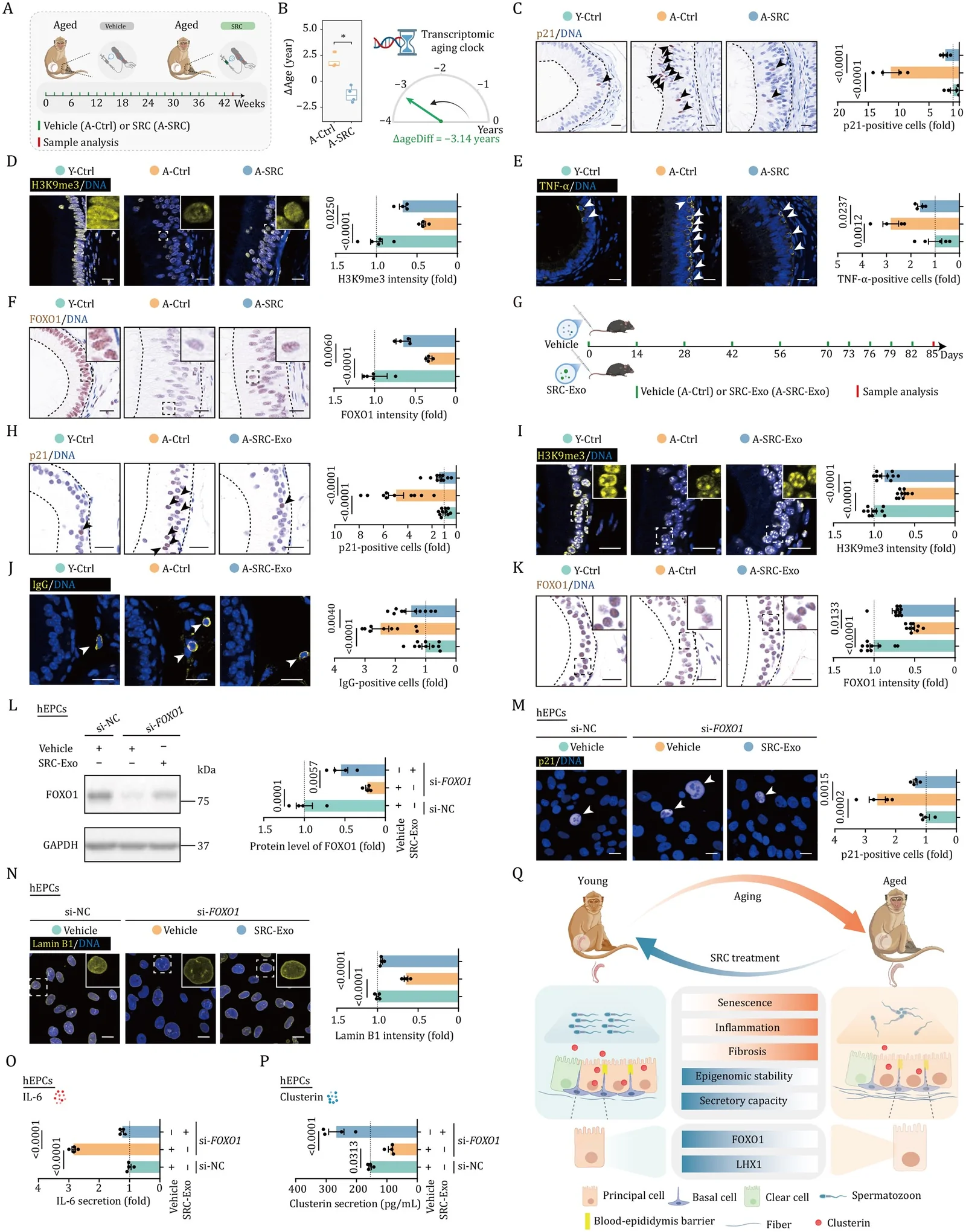
**SRC and exosome-based intervention delays epididymal aging in primates and mice**. (A) Schematic illustration of the experimental procedure for SRC and vehicle administration in aged cynomolgus monkeys. (B) Box plot shows the differences between predicted transcriptomic age and chronological ages in aged control (A-Ctrl) and aged SRC-treated (A-SRC) groups (left), and a semicircular chart illustrating the reduction of predicted transcriptomic age by SRC treatment in aged cynomolgus monkeys (right). A two-tailed Wilcoxon rank-sum test was performed. Asterisks indicate *P *< 0.05. (C) Immunohistochemical staining of p21 in the epididymis of young control (Y-Ctrl), aged control (A-Ctrl), and aged SRC-treated (A-SRC) monkeys. The numbers of p21-positive cells were quantified as fold changes (A-Ctrl vs. Y-Ctrl and A-SRC vs. A-Ctrl). The arrowheads indicate p21-positive cells. Scale bars, 20 μm. Y-Ctrl, *n *= 5; A-Ctrl, *n *= 4; A-SRC, *n *= 4. (D) Immunofluorescence examination of H3K9me3 of the epididymis of Y-Ctrl, A-Ctrl, and A-SRC monkeys. The relative intensity of H3K9me3 in epididymal epithelium was quantified as fold changes (A-Ctrl vs. Y-Ctrl and A-SRC vs. A-Ctrl). Scale bars, 20 μm. (E) Immunofluorescence examination of TNF-α in the epididymis of Y-Ctrl, A-Ctrl, and A-SRC monkeys. The numbers of TNF-α-positive cells were quantified as fold changes (A-Ctrl vs. Y-Ctrl and A-SRC vs. A-Ctrl). The arrowheads indicate TNF-α-positive cells. Scale bars, 20 μm. Y-Ctrl, *n *= 5; A-Ctrl, *n *= 4; A-SRC, *n *= 4. (F) Immunohistochemical staining of FOXO1 in the epididymis of Y-Ctrl, A-Ctrl, and A-SRC monkeys. The relative intensity of FOXO1 in epididymal epithelium was quantified as fold changes (A-Ctrl vs. Y-Ctrl and A-SRC vs. A-Ctrl). Scale bars, 20 μm. Y-Ctrl, *n *= 5; A-Ctrl, *n *= 4; A-SRC, *n *= 4. Data are presented as means ± SEM. (G) Schematic diagram of intravenous injection of SRC-derived exosomes (SRC-Exo) into aged mice. (H) Immunohistochemical staining of p21 in the epididymis of young control (Y-Ctrl), aged control (A-Ctrl), and aged SRC-Exo-treated (A-SRC-Exo) mice. The numbers of p21-positive cells were quantified as fold changes (A-Ctrl vs. Y-Ctrl and A-SRC-Exo vs. A-Ctrl). The arrowheads indicate p21-positive epididymal principal cells. Scale bars, 20 μm. Y-Ctrl, *n *= 10; A-Ctrl, *n *= 10; A-SRC-Exo, *n *= 10. (I) Immunofluorescence examination of H3K9me3 in the epididymis of Y-Ctrl, A-Ctrl, and A-SRC-Exo mice. The relative intensity of H3K9me3 in epididymal epithelium was quantified as fold changes (A-Ctrl vs. Y-Ctrl and A-SRC-Exo vs. A-Ctrl). Scale bars, 20 μm. Y-Ctrl, *n *= 10; A-Ctrl, *n *= 10; A-SRC-Exo, *n *= 10. (J) Immunofluorescence examination of IgG in the epididymis of Y-Ctrl, A-Ctrl, and A-SRC-Exo mice. The numbers of IgG-positive cells were quantified as fold changes (A-Ctrl vs. Y-Ctrl and A-SRC-Exo vs. A-Ctrl). The arrowheads indicate IgG-positive cells. Scale bars, 20 μm. Y-Ctrl, *n *= 10; A-Ctrl, *n *= 10; A-SRC-Exo, *n *= 10. (K) Immunohistochemical staining of FOXO1 in the epididymis of Y-Ctrl, A-Ctrl, and A-SRC-Exo mice. The relative intensity of FOXO1 in epididymal epithelium was quantified as fold changes (A-Ctrl vs. Y-Ctrl and A-SRC-Exo vs. A-Ctrl). Scale bars, 20 μm. Y-Ctrl, *n *= 10; A-Ctrl, *n *= 10; A-SRC-Exo, *n *= 10. (L) Western blot analysis of FOXO1 levels in si-NC- or si-*FOXO1*-treated hEPCs following treatment with vehicle or SRC-derived exosomes. GAPDH is used as the loading control. *n* = 4 for each group. (M) Immunofluorescence analysis of p21 in si-NC- or si-*FOXO1*-treated hEPCs following treatment with vehicle or SRC-derived exosomes. The arrowheads indicate p21-positive hEPCs. Scale bars, 20 μm. *n* = 4 for each group. (N) Immunofluorescence analysis of Lamin B1 in si-NC- or si-*FOXO1*-treated hEPCs following treatment with vehicle or SRC-derived exosomes. Scale bars, 20 μm. *n* = 4 for each group. (O) The secretion level of IL-6 in the culture supernatant, collected from 1 × 10^5^ hEPCs treated with si-NC or si-*FOXO1* and subsequent treatment with vehicle or SRC-Exo, was measured by ELISA, with the si-NC group serving as the control. *n* = 4 for each group. (P) The secretion level of clusterin in the culture supernatant, collected from 1 × 10^5^ hEPCs treated with si-NC or si-*FOXO1* and subsequent treatment with vehicle or SRC-Exo, was measured by ELISA, with the si-NC group serving as the control. *n* = 4 for each group. (Q) The diagram showing the monkey epididymis aging. For (C–F and H–P), data are presented as means ± SEM, one-way ANOVA with Tukey’s multiple comparisons test *P* values are indicated.

Building upon our previous finding that long-term infusion of SRCs attenuates systemic aging in NHPs (Lei et al., 2025), we investigated the potential of this intervention to specifically counteract epididymal aging. Employing an epididymal transcriptomic aging clock, we calculated that SRC treatment reduced the estimated biological age of the epididymis by 3.14 years in monkeys (Figs. 6A, 6B, S3H and S3I). Histological assessment confirmed a reduction of aging phenotypes in SRC-treated animals. This was characterized by a lower abundance of p21-positive senescent cells (Fig. 6C), improved heterochromatin stability as indicated by H3K9me3 levels (Fig. 6D), diminished infiltration of CD45^+^ immune cells, and reduced expression of the pro-inflammatory cytokine TNF-α (Figs. 6E and S3J). Notably, SRC intervention restored the age-associated downregulation of the geroprotective transcription factor FOXO1 in the epididymal epithelium (Figs. 6F and S3K).

Given the established role of exosomes in mediating the senoprotective effects of stem cells (Lei et al., 2025; Wang and Du, 2025), we next evaluated whether SRC-derived exosomes (SRC-Exo) could replicate these benefits. Repeated intravenous administration of SRC-Exo to aged mice effectively delayed epididymal aging, as shown by a reduction in p21^+^ senescent cells, an increase in heterochromatin marker H3K9me3, and a decrease in IgG accumulation (Fig. 6G–J). Mirroring the effects of SRC treatment in primates, SRC-Exo administration also restored FOXO1 expression in mouse epididymis (Fig. 6K). To determine if the protective effect was direct and cell-autonomous, we applied SRC-Exo to hEPCs rendered senescent by *FOXO1* knockdown. SRC-Exo treatment upregulated FOXO1 expression in these cells (Figs. 6L and S3L) and effectively ameliorated the senescent phenotypes. This was demonstrated by a decrease in SA-β-Gal and p21-positive cells (Figs. 6M and S3M), improved nuclear lamina integrity (Fig. 6N), suppressed secretion of the SASP factor IL-6 (Fig. 6O), and restored production of the functional marker clusterin (Fig. 6P).

Collectively, these results demonstrate that SRC intervention mitigates epididymal aging, and that this geroprotective effect is mediated, at least in part, through the exosome-dependent restoration of FOXO1 signaling and subsequent alleviation of epithelial cell senescence.

## Discussion

The age-related decline in male reproductive potential is closely linked to deficiencies in post-testicular sperm maturation, a process in which epididymal function is ­paramount. Our study provides a comprehensive, multi-dimensional dissection of primate epididymal aging, establishing epithelial senescence, chronic inflammation, and interstitial fibrosis as its defining tissue hallmarks. Through the construction of a single-nucleus transcriptomic atlas of the aging primate epididymis, we identify the functional erosion of PCs as a central event. We further delineate a molecular cascade wherein the age-associated downregulation of the longevity-linked transcription factor FOXO1 drives PC senescence, at least in part, through the diminished transcriptional activation of its downstream target *LHX1*. This newly identified FOXO1-LHX1 axis represents a critical protective pathway against epididymal aging, offering a mechanistic framework for understanding age-related functional decline and presenting potential targets for intervention (Fig. 6Q).

Our systematic analysis revealed a consistent accumulation of senescent cells across all epididymal regions with age. PCs, constituting the most abundant and functionally essential epithelial population, emerged as the primary senescent reservoir and exhibited the most profound transcriptional disturbance. This positions PC senescence as a likely instigator of broader tissue dysfunction. Senescent cells propagate local damage via the SASPs, releasing a milieu of inflammatory cytokines, chemokines, and proteases that recruit immune cells and promote pathological tissue remodeling, including fibrosis (Hou et al., 2023; Kale et al., 2020; Leng and Pawelec, 2022; Mebratu et al., 2023; Nelson et al., 2012; Schafer et al., 2017; Sun et al., 2022). Indeed, our observations of increased CD45^+^ and CD68^+^ cell infiltration, elevated TNF-α, and collagen deposition in the aged epididymis are congruent with this paradigm. These findings illustrate a vicious cycle wherein intrinsic epithelial senescence and the resultant adverse microenvironmental remodeling mutually reinforce each other, collectively driving tissue degeneration.

We pinpoint the downregulation of FOXO1 as a pivotal event triggering this cascade in PCs. FOXO family transcription factors are central regulators of cellular homeostasis, integrating signals to regulate stress resistance, metabolism, and longevity (Eijkelenboom and Burgering, 2013; Huang and Tindall, 2007; Jing et al., 2023; Liu et al., 2024, 2025; Martins et al., 2016). Our functional studies in human primary epididymal epithelial cells demonstrate that FOXO1 deficiency is sufficient to induce a comprehensive senescent state, characterized by cell cycle arrest, loss of nuclear and heterochromatin integrity, SASP activation, and impaired specialized secretion. Mechanistically, we establish that FOXO1 exerts its geroprotective effect, in part, by directly transcriptionally activating *LHX1*—a factor not previously implicated in cellular senescence. The FOXO1-LHX1 axis thus represents a novel regulatory module safeguarding epididymal epithelial health, whose disintegration with age contributes to functional decline.

Notably, our interventional studies indicate that this aging process is not irreversible. Administration of SRCs or their exosomes (termed SRC-Exo) attenuated key aging phenotypes in aged animals, and directly ameliorated senescence in human PCs. These benefits were associated with the restoration of FOXO1 expression. This suggests that the geroprotective effect of SRCs likely operates through a multimodal mechanism, involving both the direct delivery of pro-homeostatic signals (via exosomes) to epithelial cells and the amelioration of the pro-inflammatory tissue microenvironment. The specific bioactive components within SRC-Exo that mediate FOXO1 upregulation and senescent cell rejuvenation warrant further investigation.

In conclusion, by mapping the cellular and molecular landscape of the aging primate epididymis at single-nucleus resolution, this work defines the inactivation of the FOXO1-LHX1 transcriptional pathway as a key driver of epithelial senescence and tissue dysfunction. Beyond identifying a new therapeutic axis for male reproductive aging, this study provides an essential reference atlas and a mechanistic foundation for future research aimed at diagnosing, mitigating, or reversing age-related decline in epididymal function and overall reproductive health.

## Supplementary Material

pwag020_Supplementary_Data

## Data Availability

The raw sequencing data of human epithelial cells reported in this paper have been deposited in the Genome Sequence Archive-Human in National Genomics Data Center, China National Center for Bioinformation, with accession number HRA014320. The raw sequencing data of animals have been deposited in the Genome Sequence Archive in National Genomics Data Center, with accession number CRA030395.

## Code availability

All the custom codes are available from the corresponding authors upon reasonable request.

## Artificial intelligence (AI)

During the writing and editing of this manuscript, the authors used ChatGPT and DeepSeek in order to check grammar and improve readability. After using these tools, the authors reviewed and edited the content as needed and take full responsibility for the content of the published article. In addition, the icon representing basal cells in Fig. 3A was generated using the AI tool Doubao and was used solely for schematic illustration purposes.

## Abbreviations

BC: basal cell
CC: clear cell
DCN: decorin
DEGs: differentially expressed genes
EC: endothelial cell
ELISA: enzyme-linked immunosorbent assay
hEPCs: human epididymal principal cells
FACS: fluorescence-activated cell sorting
FOXO1: Forkhead box protein O1
IGKC: immunoglobulin kappa light chain constant region
IL-6: interleukin-6
Mac: macrophage
PC: principal cell
SASP: Senescence-Associated Secretory Phenotype
SC: stromal cell
siRNA: small interfering RNA
snRNA-seq: single-nucleus RNA sequencing
SRCs: senescence-resistant human mesenchymal progenitor cells
T: T cell
TF: transcription factor
TNF-α: tumor necrosis factor-alpha
UMAP: uniform manifold approximation and projection
