# Supplementary material for: Restoring the FOXO1 geroprotective pathway via seno-resistant mesenchymal progenitor cells alleviates primate epididymal aging
Source: Protein Cell. 2026 Mar 24;17(9):840–57. doi: 10.1093/procel/pwag020 (PMC13521284; doi:10.1093/procel/pwag020)
Supplement: Supplementary file 1 [file pwag020_supplementary_data.zip › Supplementary_data/Supplementary data.pdf]

Supplemental materials

Methods

Experimental animals

The cynomolgus monkeys (*Macaca fascicularis*) used in this study were obtained from Southeast Asian populations (Yang et al., 2024b; Lei et al., 2025). All individuals had no history of clinical conditions or research exposure that could influence physiological aging or disease susceptibility parameters. The animals were housed at the Beijing Xieerxin Institute of Bioresources (Beijing, China), an accredited laboratory animal care facility that strictly adheres to all relevant local regulations governing animal experimentation. Environmental conditions were maintained at a constant ambient temperature (25°C) with a standardized 12-hour light/dark cycle. The monkeys were fed three times daily and provided with ad libitum access to purified water. The young group (n = 5) consisted of individuals aged 5 to 6 years, and the aged group (n = 5) included individuals aged 16 to 18 years.

In this study, epididymal tissues were obtained from an established cohort of monkeys that received long-term infusion of senescence-resistant human mesenchymal progenitor cells (SRCs), as described in a previous study (Lei et al., 2025). Following acclimatization, subjects were randomly assigned to experimental cohorts. The intervention protocol consisted of biweekly intravenous administration of suspensions of SRCs, with each dose containing 2×10^6 cells per kilogram of body weight, prepared in sterile physiological saline. This treatment regimen was maintained for a total duration of 44 weeks. The preparation of SRCs has been described in detail previsouly (Lei et al., 2025; Ning et al., 2025).

Epididymal tissues from SRC-Exo-treated mice were similarly obtained from the established cohort described in a prior study (Lei et al., 2025). In brief, the C57BL/6J mice were procured from SPF Biotech Co., Ltd. and acclimatized under controlled environmental conditions (25 ± 1°C, 50-60% humidity) with a standardized 12-hour photoperiod. Throughout the experiment, the animals had unrestricted access to sterilized water and standard laboratory feed. For exosome administration, SRC-Exo preparations containing 20 μg of protein were reconstituted in 100 μL of sterile saline and delivered through tail vein injection. Control subjects received equivalent volumes of saline vehicle.

Tissue collection

The collection of cynomolgus monkey epididymal tissues was conducted following standard procedures for tissue sampling and preservation. Bilateral epididymides were harvested, with portions of the left epididymis fixed in 4% paraformaldehyde (PFA), followed by graded ethanol dehydration, paraffin infiltration, and embedding. Additional tissue samples were cryoprotected in 30% sucrose overnight and

subsequently embedded in optimal cutting temperature (O.C.T.) compound. Both paraffin-embedded and cryosectioned tissues were used for subsequent histological analyses. The right epididymal tissues were reserved for single-nucleus RNA sequencing (snRNA-seq) and additional RNA and protein analyses.

Masson's trichrome staining

Masson's trichrome staining was performed in accordance with the manufacturer's protocol (Solarbio, G1346). Paraffin-embedded tissue samples were sectioned into 5 μm-thick slices, baked overnight at 60 °C, and then deparaffinized with xylene for 30 minutes. The sections were rehydrated through a graded ethanol series (100%, 100%, 95%, 95%, 80%, and 75%) and then immersed in distilled water for 10 minutes. Subsequently, the sections were incubated in a potassium dichromate solution at 60 °C for 1 hour, followed by a 10-minute rinse under running tap water. Staining was carried out sequentially with iron hematoxylin for 5 minutes, Ponceau-acid fuchsin solution for 10 minutes, differentiation in phosphomolybdic-phosphotungstic acid solution for 10 minutes, and aniline blue staining for 5 minutes. After three washes with distilled water, the sections were differentiated in 1% acetic acid solution for 2 minutes. Dehydration was conducted through a graded ethanol series (50%, 75%, 85%, 95%, and 100%), followed by clearing in xylene. Finally, the sections were mounted with neutral resin. Image acquisition was performed using the PerkinElmer Vectra Polaris imaging system.

Immunofluorescence staining

Immunofluorescence staining was performed as previously described (Sun et al., 2023b; Ma et al., 2024; Yang et al., 2024a; Ding et al., 2025; Ma et al., 2025; Zhu et al., 2025). For cell immunofluorescence staining, the cells were fixed with 4% PFA for 10 minutes at room temperature (RT) and then washed with PBS, followed by permeabilization with 0.4% Triton X-100 for 5 minutes. For tissue immunofluorescence staining, after deparaffinization and rehydration, the tissues were subjected to antigen retrieval in sodium citrate buffer (pH 6.0) for 20 minutes, followed by washing with distilled water. The tissues were permeabilized with 0.4% Triton X-100 for 15 minutes. After washing with PBS, the tissues were blocked with 10% donkey serum in PBS for 1 hour and incubated overnight at 4°C with the primary antibody. Following washing with PBS, the secondary antibody and Hoechst 33342 (Thermo Fisher Scientific) were added and incubated at RT in the dark for 1 hour. After washing with PBS, the tissues were mounted using a mounting medium. Images were acquired with the Zeiss LSM 980 confocal system and subsequently analyzed with ImageJ.

The antibodies employed for immunofluorescence staining in this study are listed below: anti-H3K9me3 (Abcam, ab8898, 1:400), anti-Lamin B1 (Abcam, ab16048, 1:200), anti-CD68 (Abcam, ab955, 1:300), anti-CD45 (Abcam, ab10558, 1:300), anti-IGKC (Proteintech, 14678-1-AP, 1:200), anti-IgG (Huabio, HA1013, 1:800), anti-Decorin (Proteintech, 14667-1-AP, 1:200), anti-TNF-α (Proteintech, 26162-1-AP,

1:200), anti-Cytokeratin18 (Abcam, ab668, 1:200), anti-Androgen Receptor (AR) (Abcam, ab133273, 1:200), anti-Ki67 (ZSGB-bio, ZM-0166, 1:500), and anti-p21 (Cell Signaling Technology, 2947S, 1:200). Secondary antibodies used were the following: donkey anti-mouse-AF488 (Thermo Fisher, A21202, 1:500), donkey anti-mouse-AF568 (Thermo Fisher, A10037, 1:500), donkey anti-rabbit-AF488 (Thermo Fisher, A21206, 1:500), donkey anti-rabbit-AF568 (Thermo Fisher, A10042, 1:500), and donkey anti-goat AF488 (Thermo Fisher, A11055, 1:500).

Senescence-associated β-galactosidase (SA-β-Gal) staining

SA-β-Gal staining was performed according to previously published protocols (Hu et al., 2024; Ma et al., 2024). Frozen tissue sections or cells were fixed for 10 minutes with a mixture of 2% formaldehyde and 0.2% glutaraldehyde in PBS, followed by washing three times with PBS. SA-β-Gal staining solution was prepared, containing 5 mM potassium ferricyanide, 5 mM potassium ferrocyanide, 1 mg/mL X-gal, 150 mM NaCl, and 2 mM $MgCl_2$. Samples were incubated in this solution at 37°C in the dark overnight (tissues) or 8 hours (cells). After incubation, the staining solution was discarded, and the samples were washed three times with PBS. Images were acquired with a Nikon microscope, and data analysis was performed with ImageJ software.

Immunohistochemistry staining

After paraffin embedding, tissue sections were deparaffinized in xylene for 30 minutes and subsequently rehydrated through a graded ethanol series (100%, 100%, 95%, 95%, 85%, and 75%). The sections were then immersed in distilled water for 10 minutes. Antigen retrieval was performed using a pH 6.0 sodium citrate buffer with heating (20 minutes), and the slides were allowed to cool to RT. Following three washes with distilled water, endogenous peroxidase activity was quenched by treatment with 3% hydrogen peroxide for 10 minutes, followed by another three washes with distilled water. Permeabilization was carried out using 0.4% Triton X-100 for 15 minutes, followed by three washes with PBS. Non-specific binding was blocked with 10% donkey serum for 1 hour at RT. Primary antibody incubation was performed overnight at 4 °C. Then the sections were washed three times with PBS and incubated with a ready-to-use secondary antibody solution at RT for 1 hour. After another three PBS washes, DAB (3,3'-diaminobenzidine) substrate (Abclonal, RK50015) was applied for color development. Following visualization, the sections were washed three times in distilled water, stained with hematoxylin for 3.5 minutes, washed again, briefly differentiated in 1% hydrochloric acid ethanol for 1 second, and counterstained in 1% ammonia water for 15 seconds. Dehydration was performed through a graded ethanol series (50%, 70%, 85%, 95%, 100%, and 100%) for 5 minutes each, followed by clearing in xylene for 10 minutes. Finally, the sections were mounted using neutral resin.

The antibodies employed for immunohistochemistry staining in this study are listed below: anti-p21 (Cell Signaling Technology, 2947S, 1:200), anti-p21 (Abcam,

ab109520, 1:200), anti-FOXO1 (Cell Signaling Technology, 2880S, 1:200), and anti-FOXO1 (Proteintech, 18592-1-AP, 1:200).

Aggresome staining

Aggresome staining was carried out as previously described (Zhang et al., 2023a). Tissue sections were permeabilized with 0.4% Triton X-100 for 15 minutes, followed by three washes with PBS. Tissue sections were stained with the PROTEOSTAT®. Aggresome Detection Kit (Enzo Biochem, ENZ-51035-K100, 1:2,500) in the dark for 5 minutes, followed by decolorization with 1% acetic acid for 5 minutes. Samples were then washed three times with PBS, counterstained with Hoechst 33342 (Thermo Fisher Scientific) for 5 minutes, washed three times again with PBS, and mounted with a mounting medium. Images were captured utilizing a Zeiss LSM 980 confocal system.

Western blot analysis

Western blot analysis was conducted following the aforementioned protocol (Jing et al., 2023; Huang et al., 2024). After collection, tissues and cells are lysed with RIPA buffer (Beyotime, P0013B) and quantified with the BCA Protein Assay Kit (Dingguo Biotechnology, BCA02), then boiled at 105°C for 10 minutes and cooled to RT. Electrophoresis was performed using 10% SDS-PAGE, and the proteins were transferred to a PVDF (polyvinylidene fluoride) membrane (Merck Millipore). The membrane was blocked at RT with 5% non-fat milk, followed by incubation with the primary antibody overnight at 4°C. The next day, the membrane was washed three times with TBST and incubated with an HRP-conjugated secondary antibody at RT for 1 hour. After washing, protein bands were detected using the ChemiDoc XRS system, and band intensities were quantified with ImageJ.

The antibodies employed for Western blot in this study are listed below: anti-LHX1 (Abcam, ab229474, 1:1,000), anti-FOXO1 (Cell Signaling Technology, 2880S, 1:1,000)，anti-GAPDH (Abclonal, A19056, 1:3,000), and anti-Clusterin-α (Santa Cruz, sc-5289, 1:50). Secondary antibodies used were the following: goat anti-rabbit IgG (ZSGB-bio, ZB-2307, 1:5,000), and goat anti-Mouse IgG (ZSGB-bio, ZB-2305, 1:5,000).

Cell culture

Human epididymal principal cells were purchased from Suncell (SNP-H060) and cultured using human epididymal principal cell complete medium (Suncell, SNP-H060), followed by immunofluorescence staining to identify specific markers.

Knockdown of *FOXO1* or *LHX1* with small interfering RNA (siRNA)

Knockdown of *FOXO1* or *LHX1* in human epididymal principal cells was achieved using specific small interfering RNAs (siRNAs), with a non-targeting siRNA serving as the negative control (si-NC). For each transfection, a mixture of the respective siRNA (or the si-NC, 50 μM) and Lipofectamine RNAiMAX Reagent (Invitrogen, 13778150; 6 μL) was prepared separately in Opti-MEM (Gibco, 31985-070; 250 μL) and incubated for 5 minutes. The two solutions were then combined, thoroughly mixed for 20 minutes, and added to the cells in 6-well plates. After 8 hours of incubation, the medium was replaced with fresh culture medium. Samples were collected 96 hours post-transfection for subsequent analysis.

RNA extraction

RNA extraction was conducted following the previously described method (Zou et al., 2021). The samples were collected and treated with Trizol at room temperature for 10 minutes. Then, the samples were homogenized and transferred to 1.5 mL microcentrifuge tubes. Chloroform was added to the homogenate, followed by vigorous mixing, and the mixture was incubated at RT for several minutes. The samples were centrifuged at 12,000 rpm for 15 minutes, and the upper clear aqueous phase was carefully collected. An equal volume of isopropanol was added, mixed thoroughly, and centrifuged again at 12,000 rpm for 15 minutes. The supernatant was discarded, and the pellet was washed with 75% ethanol, followed by centrifugation. After the supernatant was removed, the pellet was dissolved in an appropriate volume of ultrapure water and stored at -80 °C until use.

Quantitative PCR with reverse transcription (RT-qPCR) analysis

RT-qPCR analysis was performed following the previously described method (Zhang et al., 2023b; Zhao et al., 2023; Hu et al., 2025). Reverse transcription was performed with a commercial kit (Vazyme, R323-01). A total of 1 μg of RNA was treated with 5 μL of gDNA Wiper Mix at 42 °C for 2 minutes to eliminate genomic DNA contamination. Subsequently, 4 μL of HiScript IV qRT SuperMix was added for reverse transcription at 37 °C for 15 minutes, followed by incubation at 85 °C for 5 seconds to terminate the reaction and synthesize cDNA. RT-qPCR was performed using the THUNDERBIRD SYBR qPCR Mix on a real-time PCR system. All gene expression data were normalized to the internal control, and relative quantification was conducted using the 2^−ΔΔCt method. Primer sequences used in this study are listed in Table S5.

Chromatin immunoprecipitation (ChIP)-qPCR

ChIP-qPCR was carried out as previously described (Zhao et al., 2022). A total of 1×10^6 human epididymal principal cells were collected, and the supernatant was discarded. The cells were washed once with PBS, followed by the addition of 8 mL of 1% formaldehyde for fixation on a shaker at RT for 10 minutes. Crosslinking was then quenched by adding glycine to a final concentration of 0.125 M, and the cells were

incubated for an additional 5 minutes at RT with gentle shaking. Cells were pelleted by centrifugation at 5,000×g for 5 minutes at 4°C, and the supernatant was removed. The pellet was washed three times with PBS, resuspended in lysis buffer, and incubated on ice for 5 minutes. Chromatin was subsequently sheared to fragments with a target peak size of 100-500 base pairs (bp) using a Covaris S220 focused-ultrasonicator. In parallel, Dynabeads Protein A magnetic beads were pre-incubated with either normal rabbit IgG (Cell Signaling Technology, 2729S) as a negative control or anti-FOXO1 antibody (Proteintech, 18592-1-AP) for 2 hours at 4 °C on a rotator. After discarding the unbound antibody solution, the cell lysate was added to the antibody-conjugated beads and incubated overnight at 4 °C with gentle rotation. The next day, bead–antibody–chromatin complexes were subjected to reverse crosslinking in a ThermoMixer at 68 °C and 1,300 rpm for 2 hours to release DNA. DNA was purified using phenol: chloroform: isoamyl alcohol (25:24:1) extraction. For DNA precipitation, 3 M sodium acetate and glycogen were added, followed by 100% ethanol, and the mixture was stored at –80 °C overnight. On the following day, DNA was pelleted by centrifugation and dissolved in nuclease-free water. The purified DNA fragments were subsequently analyzed by qPCR. The sequences of the primers used for qPCR amplification are listed in Table S5.

Dual-luciferase reporter assay

Cells were infected with lentiviruses carrying either FLAG-GAL4 or FLAG-FOXO1 expression plasmids. Two days post-infection, the cells were seeded into 24-well plates and co-transfected with 0.9 μg of reporter plasmid (pGL3-Promoter, pGL3-LHX1-Promoter (WT), or pGL3-LHX1-Promoter (Mut)) along with 0.1 μg of Renilla luciferase plasmid as an internal control, using Lipofectamine 3000 (Thermo Fisher Scientific) according to the manufacturer's instructions. The culture medium was replaced 8–12 hours after transfection. After 48 hours post-transfection, the culture medium was removed, and the cells were washed once with PBS. A total of 120 μL of Lysis Buffer was added to each well, and the plate was incubated on a shaker at RT for 10 minutes. The lysates were then transferred to 1.5 mL microcentrifuge tubes. For luciferase activity measurement, 10 μL of lysate was added to an opaque white 96-well plate, followed by 90 μL of Firefly Assay Reagent I to measure firefly luciferase activity using a luminometer. Subsequently, 90 μL of Renilla Assay Reagent II was added to quantify Renilla luciferase activity. Firefly luciferase activity was normalized to Renilla luciferase activity to account for differences in transfection efficiency.

Plasmid construction

To construct plasmids expressing FOXO1 and LHX1, cDNA from human epididymal principal cells was used as a template for PCR amplification. The amplified FOXO1 fragment was cloned into the pLE4 vector pre-digested with XbaI and SalI, while the LHX1 fragment was inserted into the pLE4 vector digested with XbaI and MluI. The

pLE4 vector expressing GAL4 was used as a control. The sequences of the primers used for PCR amplification are listed in Table S5.

Lentivirus packaging

When HEK293T cells reached approximately 90% confluence, the pLE4-GAL4 or pLE4-FOXO1 plasmid, psPAX2 plasmid (Addgene, #12260), pMD2.G plasmid (Addgene, #12259), and PEI MAX (MW 40000, PolySciences, 24765-1) were combined in Opti-MEM (Gibco, 31985-070). The mixture was vortexed thoroughly and incubated at RT for 30 minutes. Subsequently, the transfection mixture was added to the HEK293T cell culture. The supernatant was collected at 24, 48, and 72 hours post-transfection, followed by centrifugation at 19,400 rpm for 2 hours at 4°C. Finally, the viral pellet was resuspended in an appropriate volume of culture medium and stored at -80°C for future use.

Enzyme-linked immunosorbent assay (ELISA) analysis

To evaluate the level of IL-6 in human epididymal principal cells, the culture supernatant was collected and cell counting was performed. The samples were stored at -80 °C until analysis. IL-6 quantification was conducted using the ELISA MAX™ Deluxe Set Human IL-6 kit (BioLegend, 430515) according to the manufacturer's protocol. To further assess the secretory function of the human epididymal principal cells, the level of clusterin in the culture supernatant was measured using the Human Clusterin ELISA Kit (Abcam, ab174447) as per the manufacturer's instructions.

Exosome isolation

Exosomes from human SRC-conditioned medium were obtained via differential centrifugation and filtration, as previously described (Lei et al., 2025). The medium was centrifuged at 1,500 rpm for 20 minutes and passed through a 0.22-μm filter to remove cells, debris, and large vesicles. The filtrate was ultracentrifuged at 100,000 × g for 2 hours at 4 °C, and the pellet was resuspended in saline. After a fivefold dilution, a second ultracentrifugation under identical conditions was performed for washing. The final exosome pellet was suspended in saline and stored at -80 °C.

Cell exposure to exosomes

FOXO1 expression was first silenced by siRNA transfection, as previously described (Liu et al., 2026). On the following day, cells were treated with exosomes at a concentration of 20 μg/mL. The culture medium was refreshed every other day, with the addition of fresh exosomes, and samples were collected after six days of treatment.

Nuclei isolation and snRNA-seq

Frozen epididymal caput, corpus, and cauda tissues were individually homogenized, and equal numbers of nuclei were collected by flow cytometry, pooled and processed for snRNA-seq using the 10x Genomics Chromium platform (Jing et al., 2023; Hu et al., 2024; Wang et al., 2025a; Wang et al., 2025b). All procedures were conducted on ice or at 4°C. Tissue samples were first pulverized in liquid nitrogen and then homogenized in lysis buffer (60 Hz, 30 seconds, 3 times). The cell lysis buffer consisted of nuclease-free water, 0.1% Triton X-100, 5 mmol/L $MgCl_2$, 25 mmol/L KCl, 10 mmol/L Tris buffer, 250 mmol/L sucrose, 0.4 U/μL RNasin Plus RNase inhibitor (Promega, N2615), 1 μmol/L dithiothreitol, 0.2 U/μL SUPERaseIN RNase inhibitor (Invitrogen, AM2694), and 1 × protease inhibitor.The lysate was filtered through a 40-μm strainer, and nuclei were collected by centrifugation (2,500 × g, 8 minutes, 4°C) and resuspended in PBS supplemented with 0.5% BSA and RNaseOUT Recombinant Ribonuclease Inhibitor (Thermo, 10777019). Subsequently, double-positive nuclei stained with Hoechst 33342 and propidium iodide (PI) were sorted using fluorescence-activated cell sorting (FACS) (BD Influx) and counted with a dual-fluorescence cell counter (LUNA-FLTM, Logos Biosystems). Library preparation was performed following the manufacturer's standard protocol (10x Genomics), and sequencing was carried out on the Illumina NovaSeq 6000 platform.

10x Genomics snRNA-seq data processing and quality control

Raw snRNA-seq data generated using the 10x Genomics platform were aligned to the *Macaca fascicularis* reference genome (version 5.0). Barcode processing and unique molecular identifier (UMI) quantification were performed with the 10x Genomics Cell Ranger pipeline (version 6.0.2). CellBender (version 0.3.2) was used to unsupervisedly remove systematic background noise from the data (Fleming et al., 2023). Seurat R package (version 4.3.0.1) was used to perform comprehensive downstream analyses, including the removal of low-quality cells, normalization of gene expression data, dimensionality reduction, clustering, and differential expression analysis (Hao et al., 2021; Sun et al., 2023a; Geng et al., 2025; Wang et al., 2025b). Low-quality cells expressing fewer than 200 genes or more than 6,000, and exhibiting a mitochondrial gene expression ratio greater than 5% were excluded from the analysis. Doublet detection was performed using DoubletFinder (version 2.0.3) (McGinnis et al., 2019). Following quality control procedures, a total of 90,715 high-quality cells were retained for downstream analyses.

snRNA-seq data integration, clustering, and cell type annotation

To integrate the datasets and correct for batch effects, data were first normalized and scaled using the Seurat functions "NormalizeData" and "ScaleData". Principal component analysis (PCA) was then performed with "RunPCA". To correct for batch effects, the "RunHarmony" function from the Harmony package (version 1.2.1) was applied (Korsunsky et al., 2019), and the Harmony-adjusted embeddings were used in place of the original PCA embeddings for subsequent analyses. Dimensionality

reduction was achieved using “RunUMAP”, followed by clustering with “FindNeighbors” and “FindClusters”. Cluster-specific marker genes were identified using “FindAllMarkers” based on the Wilcoxon rank-sum test, applying a threshold of avg_log$_2$(fold change) > 0.5 and p_val_adj < 0.05, with all other parameters kept at default. Cell type annotation was performed based on canonical marker genes.

Bulk RNA-seq analysis

Raw paired-end RNA sequencing reads were preprocessed using Trim Galore (version 0.6.7) to remove adapter sequences and low-quality reads. The processed high-quality reads were subsequently aligned to the *Macaca fascicularis* reference genome (5.0, ensemble 91) and *Homo sapiens* (GRCh37.87 genome) using HISAT2 aligner (version 2.2.1) (Kim et al., 2019). Gene-level read quantification was performed with featureCounts (version 2.0.1) (Liao et al., 2014). Subsequent differential expression analysis was conducted in R using the DESeq2 package (version 1.34.0) (Love et al., 2014). Genes with an adjusted p-value < 0.05 (Benjamini-Hochberg correction) and |Log$_2$(fold change)| > 0.5 for monkey tissues or > 1 for human epididymal epithelial cells were considered differentially expressed.

Identification of cell type-specific differentially expressed genes

For each cell type, aging-associated differentially expressed genes (DEGs) between young and aged groups were identified using the “FindMarkers” function from the Seurat package, applying the Wilcoxon rank-sum test. DEGs were defined by a threshold of |avg_log$_2$ (fold change)| > 0.25 and p_val_adj < 0.05.

Pathway enrichment analysis

The pathway enrichment analysis of aging DEGs and cell-type-specific marker genes was performed by Metascape (version 3.5) (Zhou et al., 2019), a comprehensive web-based platform for functional annotation.

Transcriptional noise analysis

Age-associated transcriptional noise was used to evaluate the effects of aging on transcriptional stability across different cell types, following a previously described pipeline (Angelidis et al., 2019). Transcriptional noise was calculated independently for each cell type containing at least five cells in both the young and aged groups. For each cell type, cells were downsampled to equalize cell numbers between age groups, and library size downsampling was applied to control for sequencing depth differences. Transcriptional noise was defined as the Euclidean distance between each cell and the mean expression profile of the corresponding cell type within the same age group. Changes in transcriptional noise were quantified at both the cellular and sample levels by calculating the relative difference between the aged and young groups. The consistency between cellular- and sample-level transcriptional noise changes was

assessed using robust linear regression, and model significance was evaluated by a robust F-test.

Gene set score analysis

To evaluate gene set activity at the single-cell level from snRNA-seq data, we employed the R package AUCell (version 1.8.0) (Aibar et al., 2017). Classical signaling pathway gene sets were downloaded from KEGG (Kanehisa et al., 2021) and MSigDB (Liberzon et al., 2015) database. Preprocessed single-nucleus expression data were input into AUCell for analysis. “AUCell_buildRankings” was used first to rank gene expression for each cell, and subsequently “AUCell_calcAUC” was used to calculate the AUC scores to quantify gene set enrichment in each cell. The results were visualized with the ggplot2 and ggpubr R packages, and group-wise statistical comparisons of the scores were conducted using the Wilcoxon rank-sum test implemented in ggpubr (version 0.6.0). Gene sets used in this study are shown in Table S3.

Pseudotime analysis

To characterize the dynamic changes of principal cells along the developmental trajectory, pseudotime analysis was performed using Monocle2 R package (version 2.14.0) (Trapnell et al., 2014). To reduce computational complexity, principal cells were randomly downsampled to retain 10,000 cells. A Monocle CellDataSet was constructed based on the Seurat object, incorporating cell phenotypic information (phenoData) and gene feature information (featureData). The expression matrix was normalized and dispersion was estimated using the “estimateSizeFactors” and “estimateDispersions” functions. Genes expressed in at least 10 cells with an expression level greater than 0.1 were retained using the “detectGenes” function. Ordering genes were identified by the “differentialGeneTest” function ($q < 0.01$) based on the Seurat-derived clustering results of principal cells. Dimensionality reduction was then performed using the DDRTree method (reduceDimension, method = “DDRTree”), followed by pseudotime ordering of cells with the “orderCells” function to reconstruct the developmental trajectory and identify trajectory states. Trajectories were visualized using the “plot_cell_trajectory” function.

Cell-cell communication analysis

iTALK tool (https://github.com/Coolgenome/iTALK) was used to conduct cell-cell communication analysis on snRNA-seq data to quantitatively infer and analyze intercellular communication networks. Differentially expressed ligands and receptors within each cell type were identified using the “DEG” function with thresholds of $|\log_2 \text{(fold change)}| \geq 0.25$ and q-value < 0.05. The “FindLR” function was used to identify potential receptor-ligand pairs, and the “LRPlot function” was used to visualize the results of the communication network.

Transcriptomic aging clock analysis

In previous studies (Lei et al., 2025), elderly monkeys were randomly assigned to the control (A-Ctrl) and SRC (A-SRC) groups, receiving biweekly injections of vehicle (A-Ctrl) or SRCs (A-SRC) for 44 weeks. To evaluate the protective effects of SRCs on the NHP epididymis, we constructed a transcriptomic aging clock based on epididymal transcriptomes from macaques across multiple age ranges (Lei et al., 2025). To construct a tissue-specific transcriptomic aging clock, we first identified genes significantly correlated with age using Pearson correlation analysis and selected those with high correlation as features for the aging clock. The expression matrix of these age-related genes was used as input data, while the chronological ages of control monkeys across multiple age groups served as the training labels. The aging clock was constructed using an ElasticNet regression model (Yang et al., 2024b; Zheng et al., 2024; Lei et al., 2025). 10-fold cross-validation was performed with the cv.glmnet function from the R package glmnet (version 4.1-8) to determine the optimal alpha and lambda values within the range of 0.01-0.99, and the final model was built using the glmnet function. Using this clock, we predicted the transcriptomic age of each sample and quantified the protective effect of SRCs by calculating ΔAgeDiff, defined as the difference between the mean (predicted age – chronological age) of the A-SRC and A-Ctrl groups.

Statistics & Reproducibility

Data collection and analysis were not performed blind to experimental conditions. No animals or data points were excluded, and group allocation was carried out without systematic bias prior to experimentation. Statistical analyses were performed using GraphPad Prism software (version 9.5.1). Results were presented as means ± SEM. Bulk RNA-seq and snRNA-seq data were processed using relevant R packages. Statistical methods included two-tailed Student's *t*-test, two-tailed Wilcoxon rank-sum test and one-way ANOVA. A p-value < 0.05 was considered statistically significant. In the statistical figures, asterisks indicate levels of significance as follows: * $p < 0.05$, ** $p < 0.01$, and *** $p < 0.001$; "ns" denotes no statistical significance.

References

Aibar, S., González-Blas, C.B., Moerman, T., Huynh-Thu, V.A., Imrichova, H., Hulselmans, G., Rambow, F., Marine, J.-C., Geurts, P., and Aerts, J. (2017). SCENIC: single-cell regulatory network inference and clustering. Nature methods 14, 1083-1086.

Angelidis, I., Simon, L.M., Fernandez, I.E., Strunz, M., Mayr, C.H., Greiffo, F.R., Tsitsiridis, G., Ansari, M., Graf, E., Strom, T.M., *et al.* (2019). An atlas of the aging lung mapped by single cell transcriptomics and deep tissue proteomics. Nat Commun 10, 963.

Ding, Y., Zuo, Y., Zhang, B., Fan, Y., Xu, G., Cheng, Z., Ma, S., Fang, S., Tian, A., Gao, D., *et al.* (2025). Comprehensive human proteome profiles across a 50-year lifespan reveal aging trajectories and signatures. Cell 188, 5763-5784.e5726.

Fleming, S.J., Chaffin, M.D., Arduini, A., Akkad, A.D., Banks, E., Marioni, J.C., Philippakis, A.A., Ellinor, P.T., and Babadi, M. (2023). Unsupervised removal of systematic background noise from droplet-based single-cell experiments using CellBender. Nat Methods 20, 1323-1335.

Geng, L., Ping, J., Wu, R., Yan, H., Zhang, H., Zhuang, Y., Ning, T., Wang, J., Liang, C., and Zhang, J. (2025). Systematic profiling reveals betaine as an exercise mimetic for geroprotection. Cell.

Hao, Y., Hao, S., Andersen-Nissen, E., Mauck, W.M., 3rd, Zheng, S., Butler, A., Lee, M.J., Wilk, A.J., Darby, C., Zager, M., *et al.* (2021). Integrated analysis of multimodal single-cell data. Cell 184, 3573-3587 e3529.

Hu, J., He, Z., Hu, H., Sun, X., Che, S., Sun, S., Ma, M., Zhang, Y., Ma, S., Zhang, W., *et al.* (2025). Mechanosensor YAP orchestrates human neural rosette morphogenesis via TEAD4-LEF1 transcriptional nexus. Sci China Life Sci.

Hu, Q., Zhang, B., Jing, Y., Ma, S., Hu, L., Li, J., Zheng, Y., Xin, Z., Peng, J., Wang, S., *et al.* (2024). Single-nucleus transcriptomics uncovers a geroprotective role of YAP in primate gingival aging. Protein Cell 15, 612-632.

Huang, D., Zhao, Q., Yang, K., Lei, J., Jing, Y., Li, H., Zhang, C., Ma, S., Sun, S., and Cai, Y. (2024). CRL2APPBP2-mediated TSPYL2 degradation counteracts human mesenchymal stem cell senescence. Science China Life Sciences 67, 460-474.

Jing, Y., Zuo, Y., Yu, Y., Sun, L., Yu, Z., Ma, S., Zhao, Q., Sun, G., Hu, H., Li, J., *et al.* (2023). Single-nucleus profiling unveils a geroprotective role of the FOXO3 in primate skeletal muscle aging. Protein Cell 14, 497-512.

Kanehisa, M., Furumichi, M., Sato, Y., Ishiguro-Watanabe, M., and Tanabe, M. (2021). KEGG: integrating viruses and cellular organisms. Nucleic Acids Res 49, D545-d551.

Kim, D., Paggi, J.M., Park, C., Bennett, C., and Salzberg, S.L. (2019). Graph-based genome alignment and genotyping with HISAT2 and HISAT-genotype. Nature biotechnology 37, 907-915.

Korsunsky, I., Millard, N., Fan, J., Slowikowski, K., Zhang, F., Wei, K., Baglaenko, Y., Brenner, M., Loh, P.R., and Raychaudhuri, S. (2019). Fast, sensitive and accurate integration of single-cell data with Harmony. Nat Methods 16, 1289-1296.

Lei, J., Xin, Z., Liu, N., Ning, T., Jing, Y., Qiao, Y., He, Z., Jiang, M., Yang, Y., Zhang, Z., *et al.* (2025). Senescence-resistant human mesenchymal progenitor cells counter aging in primates. Cell 188, 5039-5061.e5035.

Liao, Y., Smyth, G.K., and Shi, W. (2014). featureCounts: an efficient general purpose program for assigning sequence reads to genomic features. Bioinformatics 30, 923-930.

Liberzon, A., Birger, C., Thorvaldsdóttir, H., Ghandi, M., Mesirov, J.P., and Tamayo, P. (2015). The Molecular Signatures Database (MSigDB) hallmark gene set collection. Cell Syst 1, 417-425.

Liu, L., Zheng, Z., You, W., Yang, P., Wen, Y., Qiao, Y., Ma, S., Zhang, H., Zhang, S., and Xu, G. (2026). Vitamin C inhibits ACSL4 to alleviate ferro-aging in primates. Cell Metabolism.

Love, M.I., Huber, W., and Anders, S. (2014). Moderated estimation of fold change and dispersion for RNA-seq data with DESeq2. Genome biology 15, 550.

Ma, S., Ji, Z., Zhang, B., Geng, L., Cai, Y., Nie, C., Li, J., Zuo, Y., Sun, Y., Xu, G., *et al.* (2024). Spatial transcriptomic landscape unveils immunoglobin-associated senescence as a hallmark of aging. Cell 187, 7025-7044.e7034.

Ma, Y., Zhang, J., Liu, H., Li, D., Guo, S., Han, J., Wang, L., Yu, S., Su, X., and Gao, Y. (2025). Spatiotemporal characterization of disease-associated neurons in the entorhinal cortex-hippocampal circuit during Alzheimer's disease progression. Protein & Cell 16, 799-814.

McGinnis, C.S., Murrow, L.M., and Gartner, Z.J. (2019). DoubletFinder: Doublet Detection in Single-Cell RNA Sequencing Data Using Artificial Nearest Neighbors. Cell Syst 8, 329-337 e324.

Ning, T., Lei, J., Jiang, X., Sun, S., Zheng, F., Zhao, Q., Ma, S., Zhang, W., Qu, J., and Liu, G.-H. (2025). Genetically Engineered Senescence-Resistant Human Mesenchymal Progenitor Cells Promote Spinal Cord Injury Repair. Life Medicine, lnaf038.

Sun, G., Zheng, Y., Fu, X., Zhang, W., Ren, J., Ma, S., Sun, S., He, X., Wang, Q., Ji, Z., *et al.* (2023a). Single-cell transcriptomic atlas of mouse cochlear aging. Protein Cell 14, 180-201.

Sun, S., Li, J., Wang, S., Li, J., Ren, J., Bao, Z., Sun, L., Ma, X., Zheng, F., Ma, S., *et al.* (2023b). CHIT1-positive microglia drive motor neuron ageing in the primate spinal cord. Nature 624, 611-620.

Trapnell, C., Cacchiarelli, D., Grimsby, J., Pokharel, P., Li, S., Morse, M., Lennon, N.J., Livak, K.J., Mikkelsen, T.S., and Rinn, J.L. (2014). The dynamics and regulators of cell fate decisions are revealed by pseudotemporal ordering of single cells. Nat Biotechnol 32, 381-386.

Wang, J., Wang, J., Zhang, Y., Xiong, C., Liu, S.-A., Kong, Y., Zhou, J., and Wang, X. (2025a). Identifying vascular stiffening-sensitive macrophages through integration of single-cell transcriptomics and imaging flow cytometry. Biophysics Reports 11, 393.

Wang, M., He, Z., Wang, A., Sun, S., Li, J., Liu, F., Li, C., Yang, C., Lei, J., Yu, Y., *et al.* (2025b). Single-nucleus transcriptomics decodes the link between aging and lumbar disc herniation. Protein Cell 16, 667-684.

Yang, S., Liu, C., Jiang, M., Liu, X., Geng, L., Zhang, Y., Sun, S., Wang, K., Yin, J., Ma, S., *et al.* (2024a). A single-nucleus transcriptomic atlas of primate liver aging uncovers the pro-senescence role of SREBP2 in hepatocytes. Protein Cell 15, 98-120.

Yang, Y., Lu, X., Liu, N., Ma, S., Zhang, H., Zhang, Z., Yang, K., Jiang, M., Zheng, Z., Qiao, Y., *et al.* (2024b). Metformin decelerates aging clock in male monkeys. Cell 187, 6358-6378.e6329.

Zhang, H., Li, J., Yu, Y., Ren, J., Liu, Q., Bao, Z., Sun, S., Liu, X., Ma, S., Liu, Z., *et al.* (2023a). Nuclear lamina erosion-induced resurrection of endogenous retroviruses underlies neuronal aging. Cell Rep 42, 112593.

Zhang, Y., Zheng, Y., Wang, S., Fan, Y., Ye, Y., Jing, Y., Liu, Z., Yang, S., Xiong, M., Yang, K., *et al.* (2023b). Single-nucleus transcriptomics reveals a gatekeeper role for FOXP1 in primate cardiac aging. Protein Cell 14, 279-293.

Zhao, H., Ji, Q., Wu, Z., Wang, S., Ren, J., Yan, K., Wang, Z., Hu, J., Chu, Q., Hu, H., *et al.* (2022). Destabilizing heterochromatin by APOE mediates senescence. Nat Aging 2, 303-316.

Zhao, Q., Jing, Y., Ma, S., Zhang, W., Qu, J., Wang, S., and Liu, G.-H. (2023). An efficient protocol for studying human pluripotent stem cell-derived myotube senescence. Biophysics Reports 9, 232-240.

Zheng, Z., Li, J., Liu, T., Fan, Y., Zhai, Q.C., Xiong, M., Wang, Q.R., Sun, X., Zheng, Q.W., Che, S., *et al.* (2024). DNA methylation clocks for estimating biological age in Chinese cohorts. Protein Cell 15, 575-593.

Zhou, Y., Zhou, B., Pache, L., Chang, M., Khodabakhshi, A.H., Tanaseichuk, O., Benner, C., and Chanda, S.K. (2019). Metascape provides a biologist-oriented resource for the analysis of systems-level datasets. Nat Commun 10, 1523.

Zhu, Y., Liu, J., Wang, J., Ding, S., Qiu, H., Chen, X., Guo, J., Wang, P., Zhang, X., and Zhang, F. (2025). Integrative transcriptomic and epigenomic analysis identifies BCL6B as a novel regulator of human pluripotent stem cell to endothelial differentiation. Protein & Cell 16, 985-990.

Zou, Z., Long, X., Zhao, Q., Zheng, Y., Song, M., Ma, S., Jing, Y., Wang, S., He, Y., Esteban, C.R., *et al.* (2021). A Single-Cell Transcriptomic Atlas of Human Skin Aging. Dev Cell 56, 383-397.e388.

Figure S1

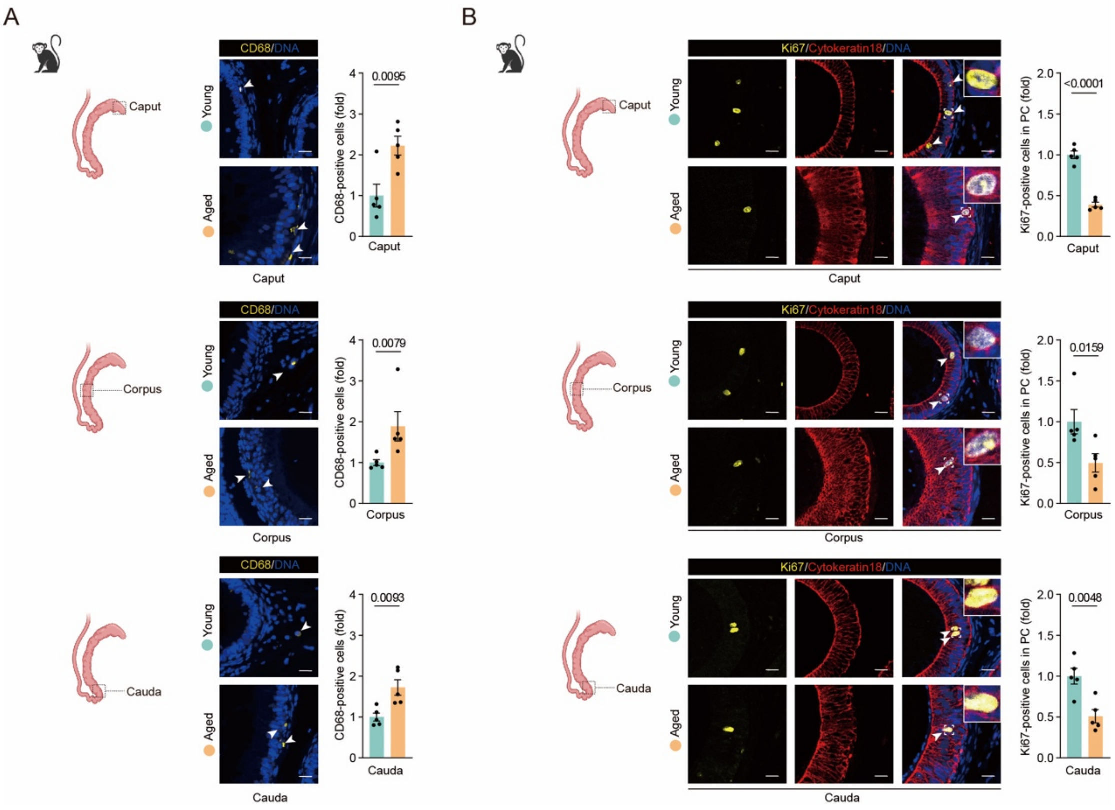

Figure S1 Phenotypic characterization of NHP epididymal aging.

(A) CD68 immunostaining in the caput, corpus, and cauda epididymis of young and aged monkeys. The numbers of CD68-positive cells were quantified as fold changes (aged vs. young). The arrowheads indicate CD68-positive cells. Scale bars, 20 µm. *n* = 5 monkeys for each group.

(B) Ki67 and cytokeratin18 immunostaining in the caput, corpus, and cauda epididymis of young and aged monkeys. The Ki67-positive cells in Cytokeratin18 were quantified as fold changes (aged vs. young). The arrowheads indicate cells that are double-positive for Ki67 and cytokeratin 18. Scale bars, 20 µm. *n* = 5 monkeys for each group.

For A-B, data are presented as means ± SEM. Two-tailed Student's t-test (for normally distributed data) or two-tailed Wilcoxon rank-sum test (for non-normally distributed data) are used.

Figure S2

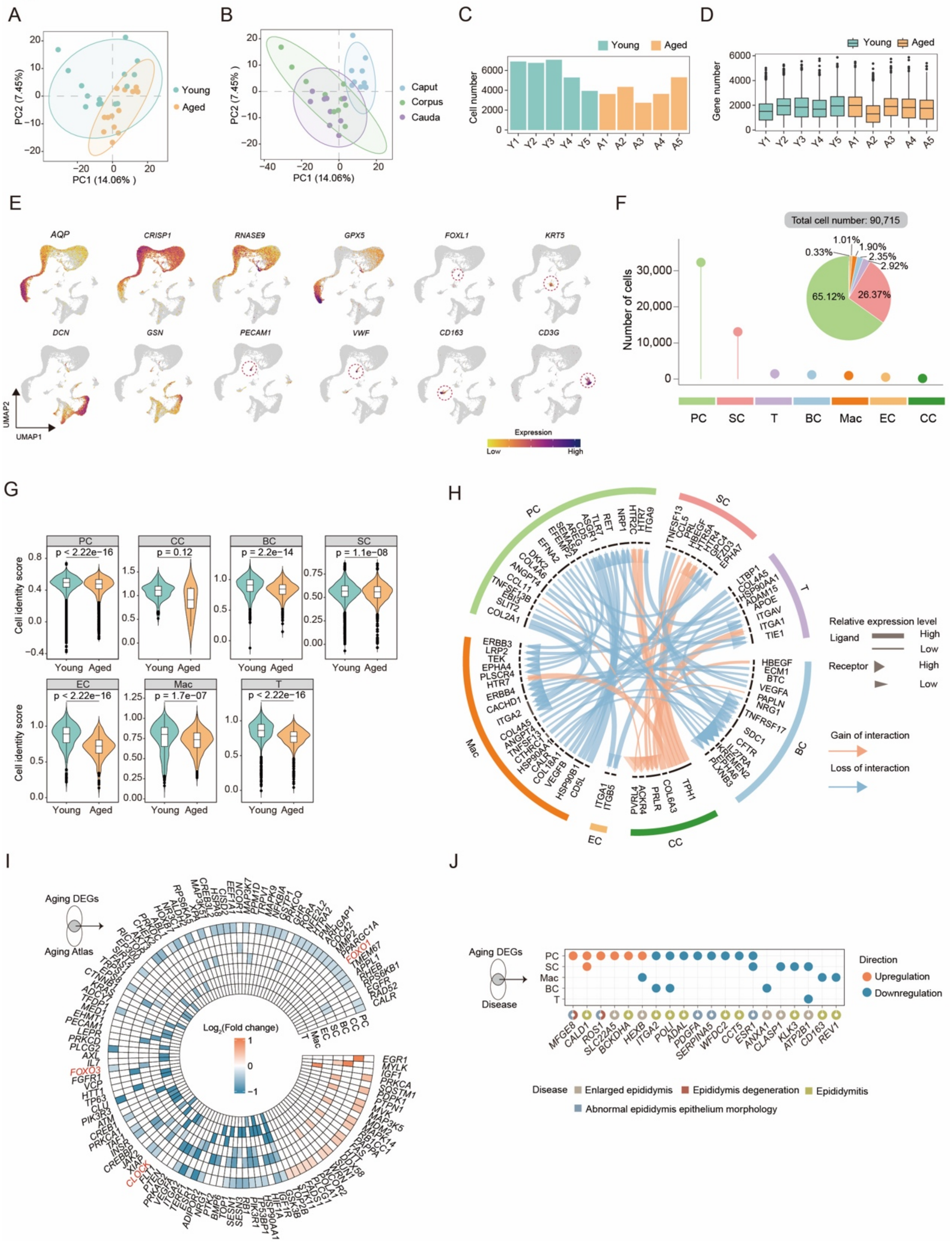

Figure S2 Single-nucleus transcriptional profiling of NHP epididymal aging.

(A) Principal component analysis shows the distribution of samples from young and aged groups based on gene expression profiles from bulk RNA-seq data of the epididymis. Each dot represents one sample, and ellipses indicate the 95% confidence interval for each group.

(B) Principal component analysis shows sample clustering according to epididymal regions based on bulk RNA-seq data of the epididymis. Each dot represents one sample, and ellipses indicate the 95% confidence interval for each region.

(C) Bar plot shows the numbers of cells detected in different samples from the snRNA-seq data of cynomolgus monkey epididymal tissues.

(D) Box plot shows the numbers of genes detected in different samples from the snRNA-seq data of cynomolgus monkey epididymal tissues.

(E) Feature plots shows the expression levels of selected marker genes of different cell types from cynomolgus monkey snRNA-seq data.

(F) Cell type composition of the epididymal snRNA-seq data. The lollipop plot displays the number of cells per identified cell type, and the pie chart shows their relative proportions.

(G) Violin plots shows cell identity scores of different cell types in the epididymis of young and aged cynomolgus monkeys.

(H) Circos plot displays ligand-receptor interactions across distinct epididymal cell types.

(I) Circular heatmap shows up- and down-regulated aging DEGs overlapping with genes annotated in the Aging Atlas database across different cell types.

(J) Dot plot shows the overlap of aging-related DEGs with epididymal disease-associated genes across different cell types (top). Donut charts depict the specific epididymal diseases associated with each overlapping gene (bottom).

Figure S3

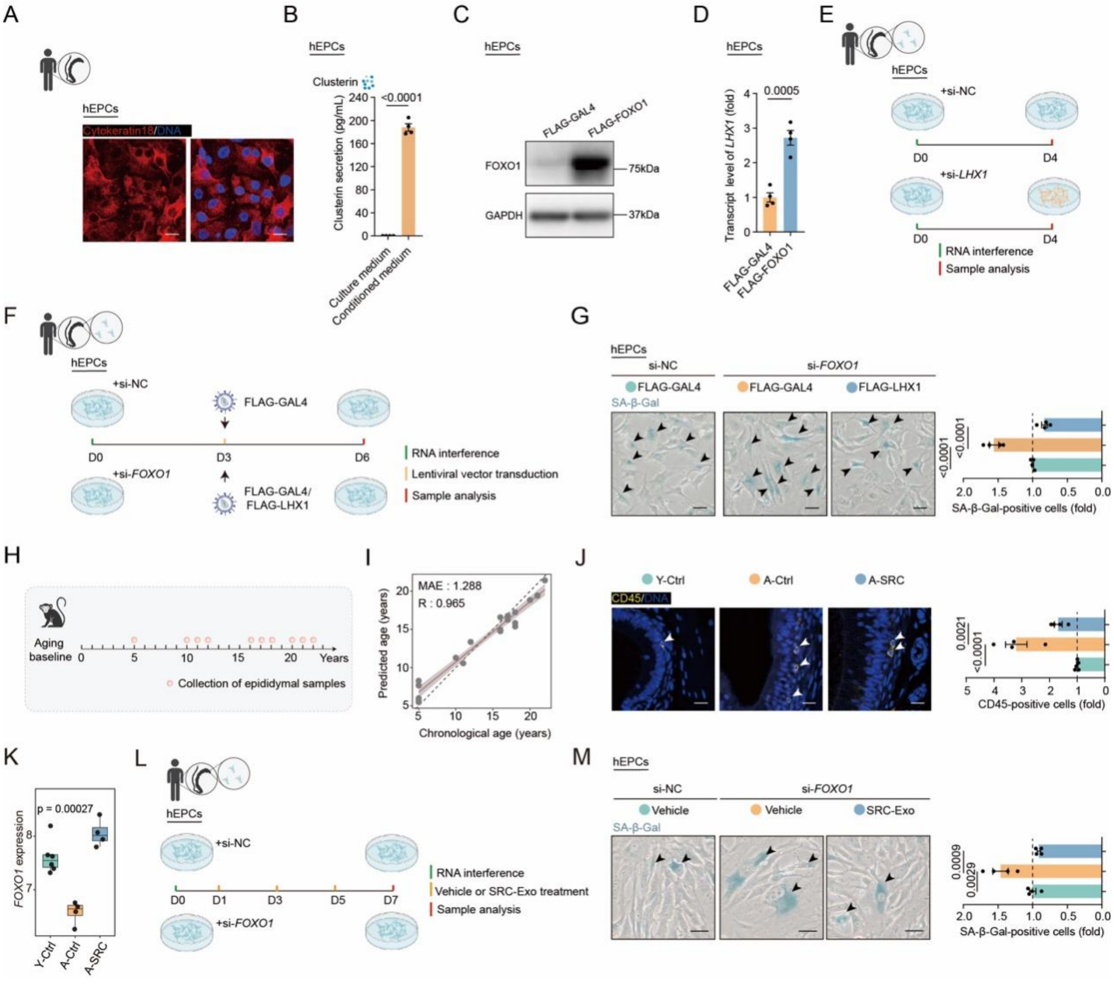

Figure S3 FOXO1 acts as a protective transcription factor in NHP epididymal aging.

(A) Immunofluorescence examination of cytokeratin 18 in human epididymal principal cells (hEPCs). Scale bars, 20 μm.

(B) The secretion level of clusterin in the culture supernatant, collected from 1×10^5 hEPCs, was measured by ELISA, with cell-free culture medium serving as the negative control. n = 4 for each group.

(C) Western blot analysis of FOXO1 in hEPCs transduced with lentiviruses expressing FLAG-GAL4 or FLAG-FOXO1.

(D) Transcript levels of *LHX1* in hEPCs transduced with lentiviruses expressing FLAG-GAL4 or FLAG-FOXO1. n = 4 for each group.

(E) The schematic illustrates the culture and treatment scheme for hEPCs subjected to si-NC or si-*LHX1* transfection, with detailed experimental conditions provided in the figure.

(F) The schematic illustrates the sequential treatment scheme for hEPCs. Following transfection with si-NC or si-*FOXO1*, the cells were transduced with lentiviruses expressing FLAG-GAL4 or FLAG-LHX1. Specific experimental conditions are detailed in the figure.

(G) SA-β-Gal staining in si-NC or si-*FOXO1* hEPCs transduced with lentiviruses expressing FLAG-GAL4 or FLAG-LHX1. The arrowheads indicate SA-β-Gal-positive cells. Scale bars, 20 μm. n = 4 for each group.

(H) Schematic illustration of epididymal sample collection across ages in cynomolgus monkeys. The timeline depicts the aging baseline, with red dots marking the specific ages of sample collection. Sample sizes at each age point were as follows: 6 monkeys at 5 years; 1 monkey each at 10, 11, 12, 20, and 22 years; 2 monkeys at 17 and 21 years; and 3 monkeys at 16 and 18 years.

(I) Dot plot shows the relationship between predicted age and chronological age in baseline animals, based on an aging clock model constructed from bulk RNA-seq data of the cynomolgus monkey epididymis. MAE, mean absolute error.

(J) Immunofluorescence examination of CD45 in the epididymis of young control (Y-Ctrl), aged control (A-Ctrl), and aged SRC-treated (A-SRC) monkeys. The numbers of CD45-positive cells were quantified as fold changes (A-Ctrl vs. Y-Ctrl and A-SRC vs. A-Ctrl). The arrowheads indicate CD45-positive cells. Scale bars, 20 μm. Y-Ctrl, n = 5; A-Ctrl, n = 4; A-SRC, n = 4.

(K) Box plot illustrates *FOXO1* transcriptional levels in the epididymis of monkeys in the Y-Ctrl, A-Ctrl and A-SRC groups. Two-tailed Wilcoxon rank-sum test was used.Y-Ctrl, n = 5; A-Ctrl, n = 4; A-SRC, n = 4.

(L) This schematic illustrates the sequential treatment protocol for hEPCs. Following initial transfection with si-NC or si-*FOXO1*, the cells were subsequently treated with either vehicle or SRC-derived exosomes (SRC-Exo). Specific experimental conditions are detailed in the figure.

(M) SA-β-Gal staining in si-NC- or si-*FOXO1*-transfected hEPCs following treatment with vehicle or SRC-Exo. The arrowheads indicate SA-β-Gal-positive cells. Scale bars, 20 μm. n = 4 for each group.

For B and D, data are presented as means ± SEM. Two-tailed Student's t-test (for normally distributed data) or two-tailed Wilcoxon rank-sum test (for non-normally distributed data) are used. For G, J and M, data are presented as means ± SEM, one-way ANOVA with Tukey's multiple comparisons test p values are indicated.

Supplementary Table Legends

Table S1. Aging-related DEGs from bulk RNA-seq or snRNA-seq data of monkey epididymis.
Table S2. Marker genes for each cell type of monkey epididymis.
Table S3. Gene sets used in this study.
Table S4. DEGs identified in hEPCs following *FOXO1* or *LHX1* knockdown.
Table S5. The sequences of primers and siRNAs used in this study.
